# Characterization of the pleural microenvironment niche and cancer transition using single-cell RNA sequencing in EGFR-mutated lung cancer

**DOI:** 10.7150/thno.85084

**Published:** 2023-08-06

**Authors:** Yu-Yuan Wu, Ya-Ling Hsu, Yung-Chi Huang, Yue-Chiu Su, Kuan-Li Wu, Chao-Yuan Chang, Chai-Tung Ong, Jia-Chen Lai, Tzu-Yen Shen, Tai-Huang Lee, Jen-Yu Hung, Ying-Ming Tsai

**Affiliations:** 1 School of Medicine, College of Medicine, Kaohsiung Medical University, Kaohsiung 807, Taiwan.; 2 Graduate Institute of Medicine, College of Medicine, Kaohsiung Medical University, Kaohsiung 807, Taiwan.; 3 Drug Development and Value Creation Research Center, Kaohsiung Medical University, Kaohsiung 807, Taiwan.; 4 Department of Pathology, Kaohsiung Medical University Hospital, Kaohsiung Medical University, Kaohsiung 807, Taiwan.; 5 Division of Pulmonary and Critical Care Medicine, Kaohsiung Medical University Hospital, Kaohsiung Medical University, Kaohsiung 807, Taiwan.; 6 Post Baccalaureate Medicine, College of Medicine, Kaohsiung Medical University, Kaohsiung 807, Taiwan.; 7 Department of Anatomy, College of Medicine, Kaohsiung Medical University, Kaohsiung 807, Taiwan.; 8 Department of Fragrance and Cosmetic Science, College of Pharmacy, Kaohsiung Medical University, Kaohsiung 807, Taiwan.; 9 School of Medicine, College of Medicine, Taipei Medical University, Taipei 110, Taiwan.; 10 Internal Medicine, Kaohsiung Municipal Ta-Tung Hospital, Kaohsiung 807, Taiwan.

**Keywords:** Pleural metastasis, complement factor, mesothelial cell, lung cancer, ferroptosis

## Abstract

**Background:** Lung cancer is associated with a high mortality rate and often complicated with malignant pleural effusion (MPE), which has a very poor clinical outcome with a short life expectancy. However, our understanding of cell-specific mechanisms underlying the pathobiology of pleural metastasis remains incomplete.

**Methods:** We analyzed single-cell transcriptomes of cells in pleural effusion collected from patients with lung cancer and congestive heart failure (as a control), respectively. Soluble and complement factors were measured using a multiplex cytokine bead assay. The role of ferroptosis was evaluated by *GPX4* small interfering RNA (siRNA) transfection and overexpression.

**Results:** We found that the mesothelial-mesenchymal transition (MesoMT) of the pleural mesothelial cells contributed to pleural metastasis, which was validated by lung cancer/mesothelial cell co-culture experiments. The ferroptosis resistance that protected cancer from death which was secondary to extracellular matrix detachment was critical for pleural metastasis. We found a universal presence of immune-suppressive lipid-associated tumor-associated macrophages (LA-TAMs) with complement cascade alteration in the MPE of the lung cancer patients. Specifically, upregulated complement factors were also found in the MPE, and C5 was associated with poor overall survival in the lung cancer patients with epidermal growth factor receptor mutation. Plasmacytoid dendritic cells (pDCs) exhibited a dysfunctional phenotype and pro-tumorigenic feature in the primary cancer. High expression of the gene set extracted from pDCs was associated with a poor prognosis in the lung cancer patients. Receptor-ligand interaction analysis revealed that the pleural metastatic niche was aggravated by cross-talk between mesothelial cells-cancer cells/immune cells via *TNC* and *ICAM1*.

**Conclusions:** Taken together, our results highlight cell-specific mechanisms involved in the pathobiological development of pleural metastasis in lung cancer. These results provide a large-scale and high-dimensional characterization of the pleural microenvironment and offer a useful resource for the future development of therapeutic drugs in lung cancer.

## Introduction

Lung cancer remains the leading cause of cancer death globally with approximately 1.8 million deaths every year, and about 50% of patients with lung cancer develop malignant pleural effusion (MPE) during their disease course 1-3. In lung cancer, pleural metastasis, either pleural nodules or effusion, is labeled as stage IV, implying an incurable disease 4. Several previous studies have demonstrated that MPE has a negative impact on the quality of life and prognosis, with a median overall survival time of 11 months 5, 6. However, studies on pleural metastasis and its molecular mechanisms in lung cancer are largely lacking. Both pleural exfoliated tumor cells and immune cells in the thoracic cavity undergo diverse alterations during cancer development and the evolution of metastatic tumor cells may in turn modify their response to therapy 7. Therefore, precise mapping of pleural-specific metastatic features is of critical importance especially to enable the development of potential biomarkers for a clinical diagnosis as well as specific therapeutic strategies for pleural metastasis.

The pleura is a dynamic, mesothelium-lined membrane that is involved both in maintaining homeostasis and responding to pleural inflammation 8-10. The pleural microenvironment is highly complex, including stroma and heterogeneous immune cell populations 4. The evolution of pleural metastasis occurs in dynamic stages, namely an invasion-metastasis cascade in which cancer cells sequentially undergo tumor transformation, invasion, extravasation, survival in pleural fluid, and metastatic colonization in a pleural microenvironment 11, 12. Pleural mesothelial cells (PMCs) cover the surface of the thoracic cavity and lung, and play an important role in the maintenance of pleural homeostasis by controlling fluid and electrolyte transport, initiation and resolution of inflammation, leukocyte migration, fibrinolysis, and cellular signaling. Alterations in both innate and adaptive immunity have been shown to contribute to the pathogenesis of cancer metastasis 11, 13. A recent study demonstrated that macrophages exhibit immune‐suppressive activities and contribute to MPE formation in osteosarcoma 14. However, studies on pleural tissue and its immune cells are largely lacking, which is an important issue as the pleural metastatic microenvironment may be different from the primary tumor. The tumor microenvironment is critical as it contains novel molecular prognostic and therapeutic targets, and therefore elucidating its mechanisms would totally transform the clinical prognosis and drug development for pleural metastasis into reality.

Few studies have investigated the single-cell transcriptome profiles of the pleural microenvironment or explored the underlying cellular and molecular mechanisms of pleural metastasis in lung cancer at a single-cell resolution. To solve this critical issue, we performed single-cell RNA sequencing (scRNA-seq) of cells from normal lung, cells from primary lung cancer, and pleural metastatic cancer cells from patients of lung cancer with MPE. We also collected cells from the pleural fluid of patients with congestive heart failure as the control group. We characterized the changes in PMCs and cancer cells which may contribute to pleural metastasis. We found that the pleural microenvironment was mainly composed of different immune cells. Moreover, macrophage and dendritic cell (DC) subsets exerted different functions and pro-tumorigenic characteristics. Importantly, we identified several factors that were highly expressed in MPE which could be used as prognostic factors and may contribute to the dysregulation of various immune cells. Our single-cell study provides an in-depth investigation of pleural metastasis in lung cancer and identifies several targets to develop effective and preventive strategies for patients with lung cancer.

## Methods

### Patient consent and specimens

Ninety-two participants were recruited at the Division of Thoracic Surgery and Division of Pulmonary and Critical Care Medicine, Kaohsiung Medical University Hospital (KMUH), Kaohsiung, Taiwan, and the study was approved by the Institutional Review Board of the hospital (KMUH-IRB-20180023, KMUH-IRB-20200038; KMUH-IRB-E(II)-20220175). Informed consents were obtained from all of the participants. Three paired adjacent non-tumor lung and tumor tissue samples, four patients with congestive heart failure, and four patients with MPE of lung adenocarcinoma were subject to scRNA-seq for transcriptome analysis. Thirteen patients with congestive heart failure were enrolled as controls, and sixty-eight patients with MPE from lung adenocarcinoma were included for associated secreted factor analysis (Table S1-2).

### scRNA-seq and analysis

Single-cell barcoding of thawed samples and complementary DNA (cDNA) library preparation were performed according to the manufacturer's protocol (Single Cell 3′ Reagent Kits v3, 10x Genomics, USA). Quality control was maintained using a Bioanalyzer High sensitivity DNA chip (Agilent 4200 TapeStation System, Amstelveen, Netherlands) and Qubit dsDNA High Sensitivity Assay Kit (Thermo Fisher, Waltham Massachusetts, US) before library preparation. The cDNA libraries were sequenced on a NextSeq 2000 Illumina platform (Illumina, Inc., San Diego, CA). Base call files were demultiplexed into FASTQ files using Cell Ranger's (v7.1.0) mkfastq pipeline. The Read2 files were trimmed using cutadapt (v2.7), and reads shorter than 20 bp were removed. Read processing was conducted with zUMIs v2.0 pipeline, and trimmed reads were aligned to GRCh38 (GRCh38.p13) using STAR (v2.7.2a). We removed barcoded cells with <100 transcripts and cells with >20% of their transcriptome of mitochondrial origin (Figure S1A).

### InferCNV

The primary cancer and pleural cancer cells were clustered to construct a new gene-cell matrix. The somatic large-scale chromosomal CNV score of cancer cells was calculated using R package InferCNV (v1.6.0). A raw counts matrix, annotation file, and gene/chromosome position file were prepared according to data requirements (https://github.com/broadinstitute/inferCNV). Ciliated cells and alveolar type II epithelial cells (AT2) were selected to act as reference of primary lung cancer cells. Mesothelial cells were chosen as reference of pleural metastatic cancer cells. The default parameters were applied (cutoff = 0; denoise = 0.2). The CNV score was calculated as quadratic sum of CNV region.

### Bioinformatics' analysis of differential expression analysis and trajectory analysis

Signature genes were expressed in >50% of cells within either of the two subsets, and changes in differential gene expression were > 2-fold (log_2_ FC > 1). Pathway enrichment analysis examining the enriched processes in clusters was performed using the DAVID website (https://david.ncifcrf.gov/) and Ingenuity Pathway Analysis (IPA). To identify the pathways potentially linked to the identified modules of analysis, trajectory analysis was performed using Monocle 2 (v2.26.0). The survival of specific gene sets was analyzed using the Gene Set Cancer Analysis (GSCA) website (http://bioinfo.life.hust.edu.cn/GSCA/#/).

### Cell-cell interactions

To investigate cell-cell interactions in different cell types including the 5 molecular subtypes, the CellChat 15 and CelltalkDB (http://tcm.zju.edu.cn/celltalkdb/) packages were used to estimate the significance of ligand-receptor pairs in different cell clusters. Ligand-receptor pairs with p < 0.05 were considered to have significant interactions between two cell types.

### Multiplex soluble factor analysis

The levels of various soluble factors in the pleural effusion were measured using enzyme-linked immunosorbent assay (ELISA) kits for human Amphiregulin (R&D Systems), Fibronectin (R&D Systems), MMP-2 (BioLegend, USA), and Macrophage migration inhibitory factor (MIF) (R&D Systems). Other factors, including CCL3, CXCL2, CXCL8, Galectin-3, Galectin-9, amphiregulin, HB-EGF, Intracellular adhesion molecule 1 (ICAM-1), IL-1β, Resistin, Plasminogen activator inhibitor 1 (PAI-1), Tenascin C (TNC), and Vascular endothelial growth factor A (VEGFA) were assessed using a Luminex assay (R&D Systems). The levels of complement system were assessed using human complement panel 1 and 2 bead-based Multiplex Assay kits (EMD Millipore).

### Cell lines and co-culture system

Pleural mesothelial MeT-5A cells (CRL-9444™) were obtained from the American Type Culture Collection (American Type Culture Collection (ATCC), Manassas, USA) and were cultured in Medium 199 containing 1.5 g/L sodium bicarbonate, 10% fetal bovine serum (FBS), epidermal growth factor (3.3 nM), hydrocortisone (400 nM), bovine insulin (870 nM), HEPES (20 mM) and trace elements (H_2_SeO_3_, MnCl_2_, Na_2_SiO_3_, (NH_4_)_6_Mo_7_O_24_, NH_4_VO_3_, NiSO_4_ and SnCl_2_). A549 (CCL-185™) and CL1-5 cell lines were cultured in F-12K Medium and RPMI-1640 medium supplied with 10% FBS, respectively. Epidermal growth factor receptor (EGFR) mutation lung cancer cell lines NCI-H1975 (H1975, CRL-5908™) and HCC827 (CRL-2868™) were obtained from ATCC and cultured in RPMI-1640 medium supplied with 10% FBS. MeT-5A cells were co-cultured with various lung cancer cell lines using a transwell culture system (pore size, 1 µm; Corning Incorporated, Corning, NY, USA) for 24 h and 48 h. Lung cancer cells were grown in the upper chamber with MeT-5A cells in the bottom chamber. Met-5A cells cultured alone served as controls.

### Western blot

MeT-5A cells were harvested and lysed using RIPA lysis buffer (EMD Millipore) for 15 min on ice. The total proteins were isolated by centrifugation at 12,000 ×g for 15 min at 4℃, and quantified using a bicinchoninic acid protein assay kit (EMD Millipore). Thirty µg total proteins were separated on 8-10% sodium dodecyl sulphate polyacrylamide gel electrophoresis and transferred to polyvinylidene difluoride membranes. The membranes were subsequently blocked in 5% non-fat milk and then incubated with the corresponding primary antibodies, including Rabbit anti-Claudin-1 (1:1,000; cat. No. 13255, Cell Signaling Technology), anti-Snail (1:1,000; cat. No. 3879, Cell Signaling Technology), anti-Slug (1:1,000; cat. No. 9585, Cell Signaling Technology), anti-Vimentin (1: 1,000, cat. No. 550513, BD Biosciences), anti-GAPDH (1:5,000; cat. No. MAB374, EMD Millipore) antibodies at 4℃ overnight. The membranes were probed with the appropriate horseradish peroxidase (HRP)-conjugated secondary antibodies, and the signals of detected proteins were visualized using an enhanced chemiluminescent kit (EMD Millipore).

### Quantitative real-time polymerase chain reaction (qRT-PCR)

Total RNA was extracted from cells and converted to cDNA using a PrimeScriptTM RT Reagent Kit (RR037A, TaKaRa), and 50 ng cDNA per reaction was mixed with Fast SYBR Green Master Mix (4385612, Applied Biosystems). The qRT-PCR analysis was conducted using a QuantStudio 3 machine (Applied Biosystems). GAPDH was used as the endogenous control to give the tested genes a relative fold change using the 2^-ΔΔCt^ method. The primers of qRT-PCR are listed in Table S3.

### *GPX4* knockdown and overexpression

The *GPX4* expression in A549 and H1975 cells was downregulated by small interfering RNA (siRNA) transfection using a mixture of four siRNA pool for each target gene (Dharmacon, CO, USA). A non-targeting siRNA pool (Dharmacon) was used as the control. In brief, exponentially growing cells were seeded in regular growth medium without antibiotics at 50% of confluence, and the cells were transfected with siRNA (10 nM) using the commercial transfection reagent DharmaFECT 4 according to the manufacturer's instructions. The cells were then incubated to verify the knockdown efficiency after 48 h before the experiments. Cells were transfected either with control plasmid (pCMV6-AC-GFP, cat. No. PS100010) or *GPX4* plasmid (*GPX4* (GFP-tagged), cat. No. RG208065, Origene, Rockville, MD, USA), the protein level of GPX4 was validated by Western blot after 48 h post-transfection.

### The cell viability of lung cancer cells in MPE

Lung cancer cells were seeded in the pleural fluid of MPE (100%) with or without a GPX4 inhibitor for 48 h. Cell viability was assessed using WST-1 analysis.

### Immunohistochemistry (IHC)

GPX4, APOE, CD68, and ZNF331 were determined by IHC in paired primary cancer and pleural metastatic cancer from 5 samples. Clinical formalin-fixed paraffin-embedded (FFPE) specimens were obtained from the KMUH. For each specimen, a pathologist reviewed the hematoxylin and eosin stain slides. Paraffin sections were dewaxed and processed for antigen retrieval. Sections were treated with H_2_O_2_ for 10 min to quench endogenous peroxidase, pre-incubated in Protein Block (TA-060-PBQ, Thermo Fisher) solution 10 min and then exposed to anti-GPX4 (cat. No. ab125066, abcam; 1:1,000 in PBS, 0.3% Triton X-100), anti-CD68 (cat. No. ab125212, abcam, 1:100) and/or anti-APOE (cat. No. GTX64352, 1:100, GenTex, Inc., Irvine, CA, USA) anti-ZNF331 (cat. No. PA5-112799, Thermo Fisher, 1:600) antibodies for 50 min and then primary antibody amplifier Quanto (TL-060-QPB, Thermo Fisher) 10 min. After washing, the sections were incubated with HRP Polymer Quanto (TL-060-QPH) for 10 min, developed with 3,3'-Diaminobenzidine (DAB) (TA-060-QHSX and TA-002-QHCX) for 3 min and then co-stained with counterstain with hematoxylin, dehydrated, cleared in xylene and coverslipped.

### Statistical analysis

All statistical analyses were carried out using GraphPad Prism software version 9.0.0. All grouped data were summarized as mean ± standard deviation (SD). An unpaired Student's t test and one-way analysis of variance (ANOVA) were used to determine the statistical significance when comparing two groups and more than two groups, respectively. Two-tailed p values less than 0.05 were considered to be statistically significant. Kaplan-Meier curves and the log-rank test were used to compare the overall survival between two groups of patients based on the level of various factors in the pleural fluid.

## Results

### The landscape of the pleural microenvironment by single-cell-based profiling

**Figure 1:**
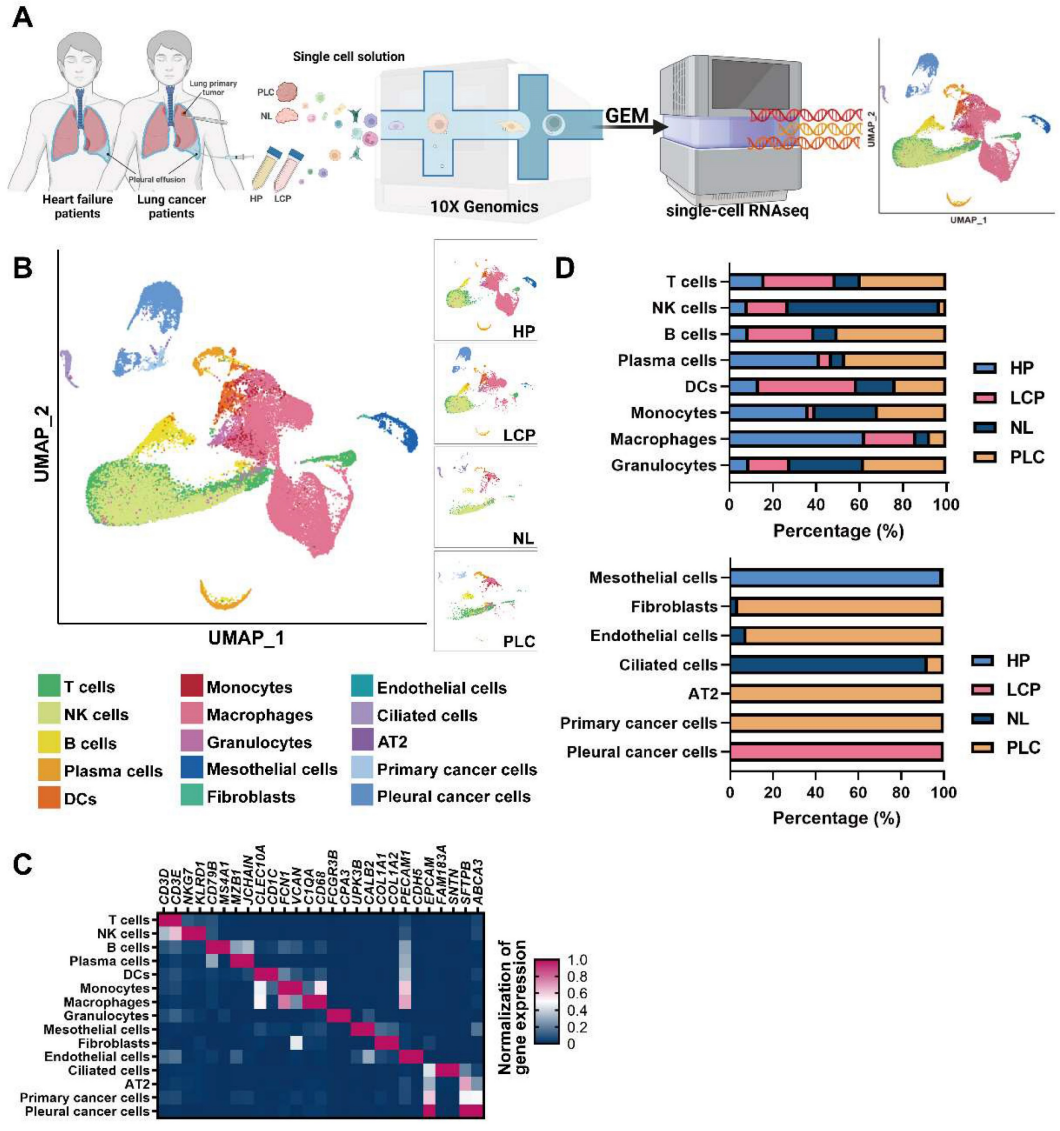
** Cell profiling in pleural fluid assessed by single-cell RNA-seq analysis.** (A) Workflow depicting the collection and processing of primary tissue and pleural fluid from patients with congestive heart failure or with lung cancer for single-cell RNA-sequencing and further study. (B) Visualization of 15 cell types on the UMAP plot. (C) The heatmap of cell markers used for cluster annotation. (D) The proportions of all cell types in the normal lung (NL), primary lung cancer (PLC), pleural fluid of heart failure (HP) and lung cancer with pleural metastasis (LCP) groups. AT2, alveolar type II epithelial cells; DCs, dendritic cells; NK cells, natural killer cells.

To resolve changes in the cancer cells and pleural microenvironment at a single cell resolution, we performed scRNA-seq of cells collected from normal lung (NL, n = 3), primary lung cancer (PLC, n = 3), and MPE from patients with lung cancer (LCP, n = 4). To explore the alternations of pleural resident cells, such as mesothelial cells and immune cells, we also harvested cells from pleural effusion caused by congestive heart failure (HP, n = 4) as the control group. Transudative effusion of HP patients caused by congestive heart failure due to hydraulic pressure with less inflammatory situation could provide the cell components in pleural space of a healthy individual. A schema of the workflow is shown in Figure 1A. Overall, 44,306 cells passed quality control (mitochondrial gene: 20%) and were eligible for subsequent analysis. To visualize the qualitative changes of cell composition in the pleura, cells were displayed in 2-D space using uniform manifold approximation and projection (UMAP) (Figure 1B and Figure S1B). The cell markers for cell annotation and the percentages of all cell types within each sample are shown in Figure 1C, Figure S2A, and Table S4. The cell counts of T cells, B cells, plasma cells, monocytes, and mast cells increased, while the number of NK cells decreased in the PLC group compared with the NL group. On the other hand, the number of T cells, NK cells, B cells, and DCs dramatically increased, whereas the number of plasma cells, monocytes, and macrophages decreased in the PLC and LCP group, compared with the HP group (Figure 1D and Figure S2B-C).

### The MesoMT of pleural mesothelial cells in the LCP group

**Figure 2:**
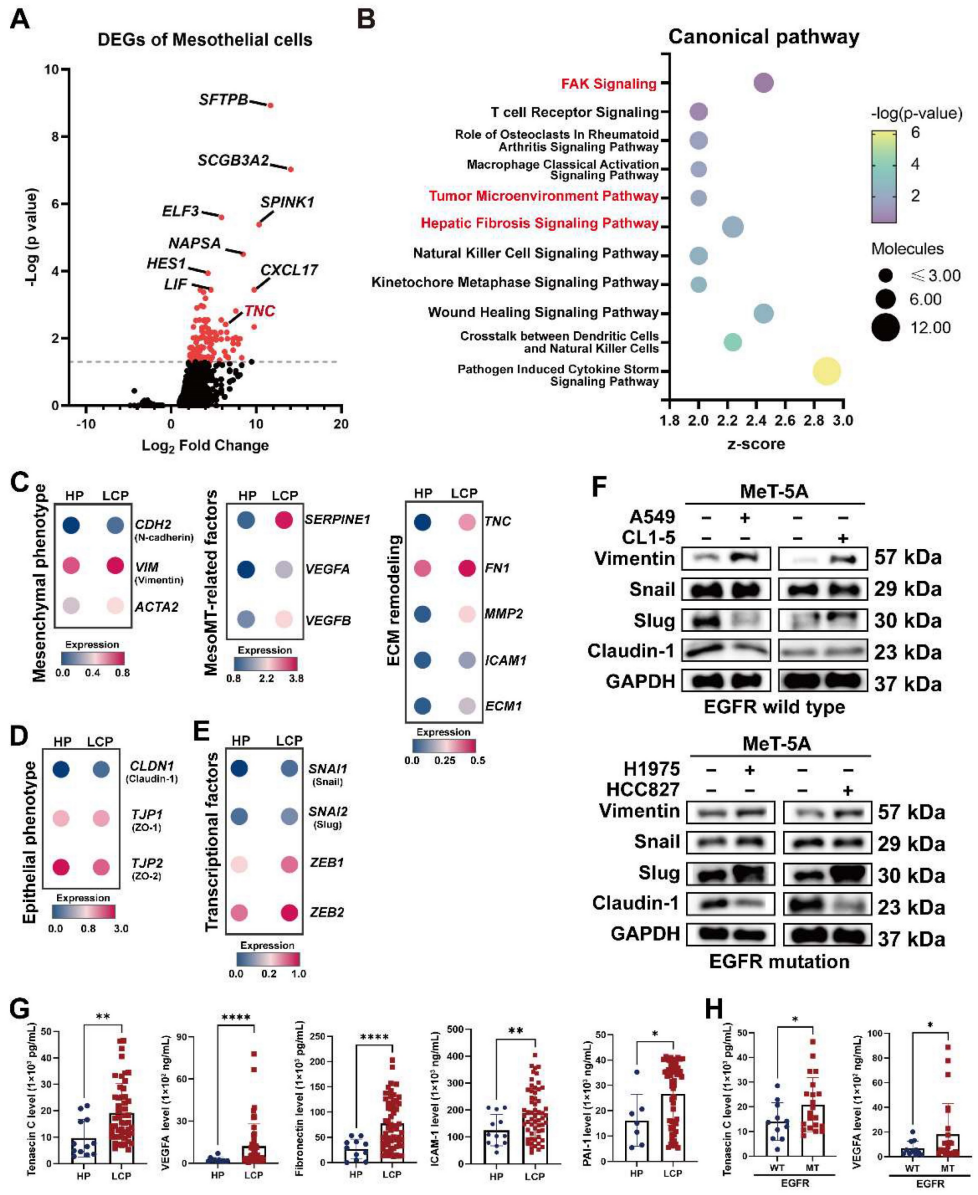
** Alterations in pleural mesothelial cells (PMCs) in pleural metastasis.** (A) The volcano plot of differentially-expressed genes (DEGs) of PMCs obtained from the HP and LCP groups. (B) The IPA canonical pathways of DEGs. Dot plot showing the related expressions of mesenchymal (C), extracellular matrix (ECM) remodeling, MesoMT related factors, epithelial (D), and transcription factors regulating the MesoMT (E). (F) Lung cancer cells stimulated MesoMT of MeT-5A mesothelial cells. (G) The levels of tenascin C, VEGFA, fibronectin, ICAM-1, and PAI-1 in the pleural fluid obtained from the HP and LCP groups. (H) The levels of tenascin C and VEGFA in pleural fluid of wild type (WT) or mutated (MT) EGFR lung cancer patients. Data in Figure 2G and 2H are presented as mean ± SD. *p < 0.05, **p < 0.01, ****p < 0.0001.

PMCs are the major cell type in the pleural space and the main cell type responsible for noxious stimuli. To investigate changes of PMCs, we evaluated the transcriptomes of PMCs (CALB1, WT1, and UPK3B-positive cells) in pleural effusion from patients with congestive heart failure or patients with lung cancer. As shown in Figure 2A and Figure S3A, 128 genes were upregulated in the PMCs of the LCP group compared with the HP group (Table S5). IPA analysis revealed that the FAK Signaling, Tumor Microenvironment Pathway, and Hepatic Fibrosis Signaling Pathway were enriched in the PMCs of the LCP group (Figure 2B and Table S6). Higher expressions of the genes involved in the Tumor Microenvironment Pathway, including *ICAM1*, *SLC2A1,* and *TNC* were found in the PMCs of the LCP group (Figure S3B). Consistent with the MesoMT and fibrosis, which are critical processes for cancer peritoneal metastasis 16, the expressions of MesoMT-related and fibrosis-related gene sets, including mesenchymal factors (*CDH2, VIM, ACTA2*), MesoMT-related factors (*SERPINE1, VEGFA, VEGFB*) and extracellular matrix (ECM) remodeling factors (*TNC, FN1, MMP2, ICAM1, ECM1*) were increased in the PMCs of the LCP group (Figure 2C). In contrast, epithelial markers* CLDN1, TJP1,* and* TJP2* were decreased in the PMCs of the LCP group (Figure 2D). The MesoMT-triggered transcription factors, including *SNAI1/2* and *ZEB1/2* were upregulated in the PMCs of the LCP group (Figure 2E). Co-culture of PMC (MeT-5A) with human lung cancer cells (EGFR wild type cancer cell lines: A549 and CL1-5; EGFR mutation cancer cell lines: H1975 and HCC827) also showed that lung cancer cells induced the MesoMT in MeT-5A cells, as supported by the upregulation of mesenchymal factors, Vimentin, and downregulation of the epithelial marker, Claudin-1 (Figure 2F). Moreover, the levels of Tenascin C, VEGFA, Fibronectin, ICAM-1, and PAI-1 were higher in the LCP group than in the HP group (Figure 2G). The levels of MMP-2 were not significantly different (Figure S3C). The levels of Tenascin C and VEGFA were higher in the MPE of EGFR-mutated lung cancer patients but not Fibronectin, ICAM-1, nor PAI-1 (Figure 2H and Figure S3D). However, all of these factors were not associated with the overall survival of the LCP group regardless of EGFR mutation status (Figure S3E-F).

### Changes in the pleural metastatic cancer cells

**Figure 3:**
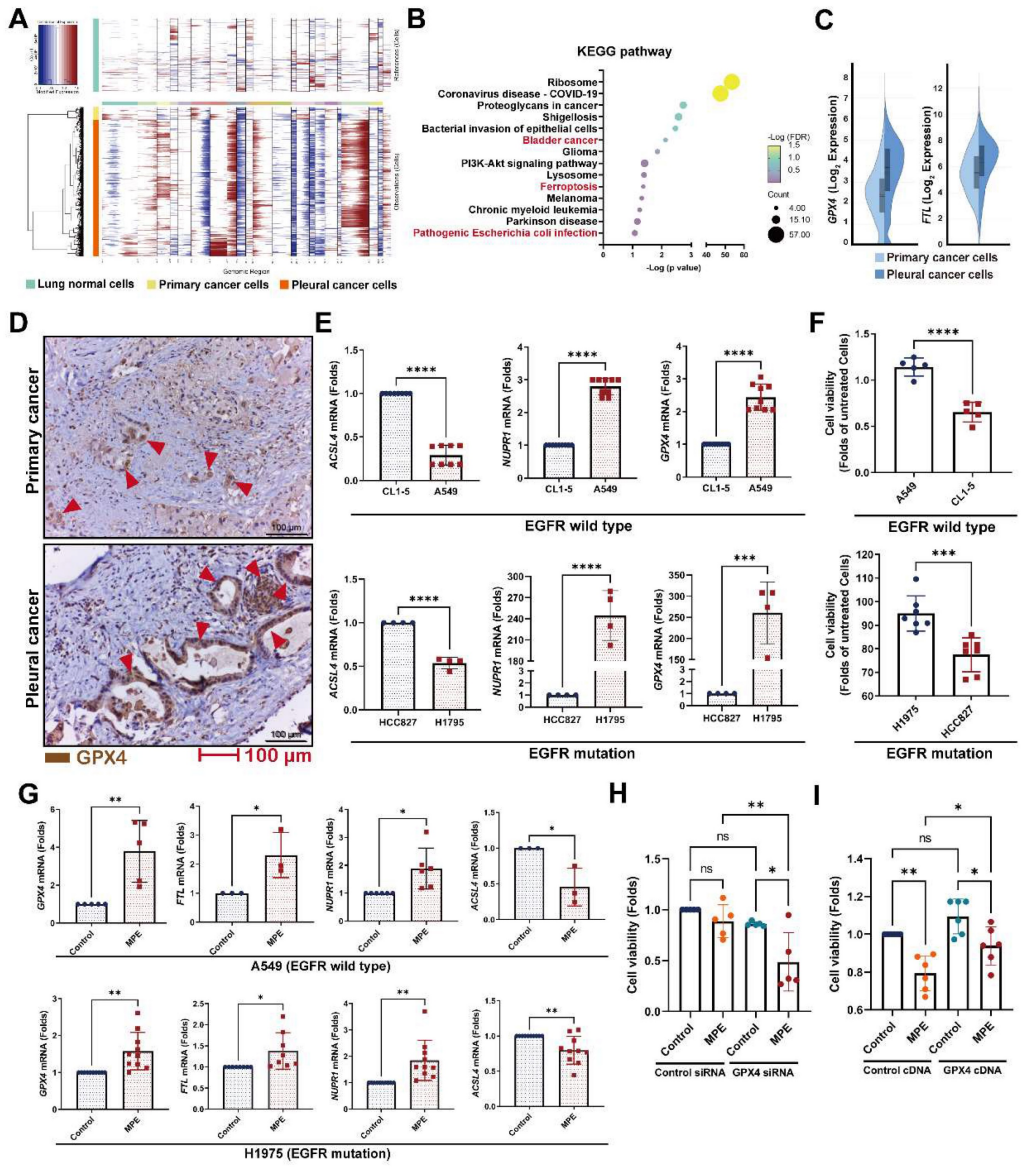
** Ferroptosis resistance contributed to pleural metastasis in lung cancer.** (A) InferCNV profiles of pleural and primary cancer cells. (B) KEGG pathway of DEGs. (C) The upregulated expressions of *GPX4* and *FTL* in pleural cancer cells. (D) IHC revealed higher levels of GPX4 protein (red arrow head) in pleural cancer than in primary cancer of a paired lung adenocarcinoma tissue. (E) The expressions of ferroptosis-related genes in A549, CL1-5, H1975 and HCC827 cells. (F) The viability of A549, CL1-5, H1975 and HCC827 cells in pleural fluid of pleural metastasis lung cancer patients. (G) MPE fluid increased expressions of *GPX4*, *FTL*, as well as *NUPR1*, and decreased the expression of *ACSL4* in A549 and H1975 cells. (H) Inhibition of GPX4 decreased the viability of H1975 cells in MPE by siRNA. (I) Overexpression of GPX4 prevented cell death induced by MPE in HCC827 cells. Lung cancer cells were cultured in the pleural fluid of MPE for 48 h. The cell viability was determined by WST-1. The expressions of various genes were measured by qRT-PCR after 24 h incubation. Data are presented as mean ± SD. ns, not significant, *p < 0.05, **p < 0.01, ***p < 0.001, ****p < 0.0001. MPE, malignant pleural effusion.

To investigate which genes contribute to pleural metastasis in lung cancer, we evaluated changes in cancer cells from the primary site and pleural effusion. First, we confirmed the malignant clusters of the primary site (lung) and pleural effusion using the “inferCNV” R package (Figure 3A and Figure S4A). We then compared differences between primary cancer cells and pleural metastatic cancer cells, and found that 275 genes (log_2_ FC≧1, p < 0.05, average reads > 0.3) were upregulated and 277 genes were downregulated in the pleural metastatic cancer cells compared with the primary cancer cells (Figure S4B and Table S7). We identified that several cancer-related pathways, including “Bladder cancer”, “Ferroptosis”, and “Pathogenic Escherichia coli infection” were associated with the LCP group in intersecting KEGG and Wiki pathway analysis (Figure 3B and Figure S4C). IPA analysis also showed that the “Immunogenic Cell Death Signaling Pathway” was inhibited (z-score = -3) in the pleural metastatic cancer cells (Figure S4D). The expressions of two ferroptosis suppressors, *GPX4* and *FTL*, were increased in the LCP group (Figure 3C). We extracted an experimentally-validated ferroptosis-related driver gene set (264 genes) from the FerrDB V2 database (http://www.zhounan.org/ferrdb/current/), and weight analysis showed that the expressions of these genes were lower in the LCP group compared with the primary cancer cells (Figure S4E-F). IHC results also showed that the level of GPX4 was higher in pleural tumor cells than in primary cancer cells in matched specimens from lung cancer patients (Figure 3D and Figure S4G). *In vitro* data showed that the expressions of ferroptosis suppressors *GPX4* and *NUPR1* were high in A549 and H1975 cells, whereas the expression of ferroptosis activator, *ACSL4*, was low compared with CL1-5 and HCC827 cells, and consequently A549 and H1975 cells had greater cell viability than CL1-5 and HCC827 cells in the MPE of LCP (Figure 3E-F). MPE collected from lung cancer patients with pleural metastasis stimulated the expressions of *GPX4*, *FTL*, and *NUPR1*, whereas decreased the expression of *ACSL4* in both A549 and H1975 cells (Figure 3G). Inhibition of *GPX4* by siRNA transfection decreased cell viability of A549 and H1975 cells in the MPE of LCP compared with lung cancer cells transfected with control siRNA (Figure 3H and Figure S4H-I), whereas ectopic expression of *GPX4* prevented cell death induced by MPE in HCC827 cells (Figure 3I). Similarly, the GPX4 chemical inhibitor (26a) also decreased viability of A549 cells in pleural fluid of MPE (Figure S4J). These data suggested that the development of ferroptosis resistance may be a critical factor allowing lung cancer cells to survive in the pleural cavity.

### Distinct populations of monocytes/macrophages in MPE

**Figure 4:**
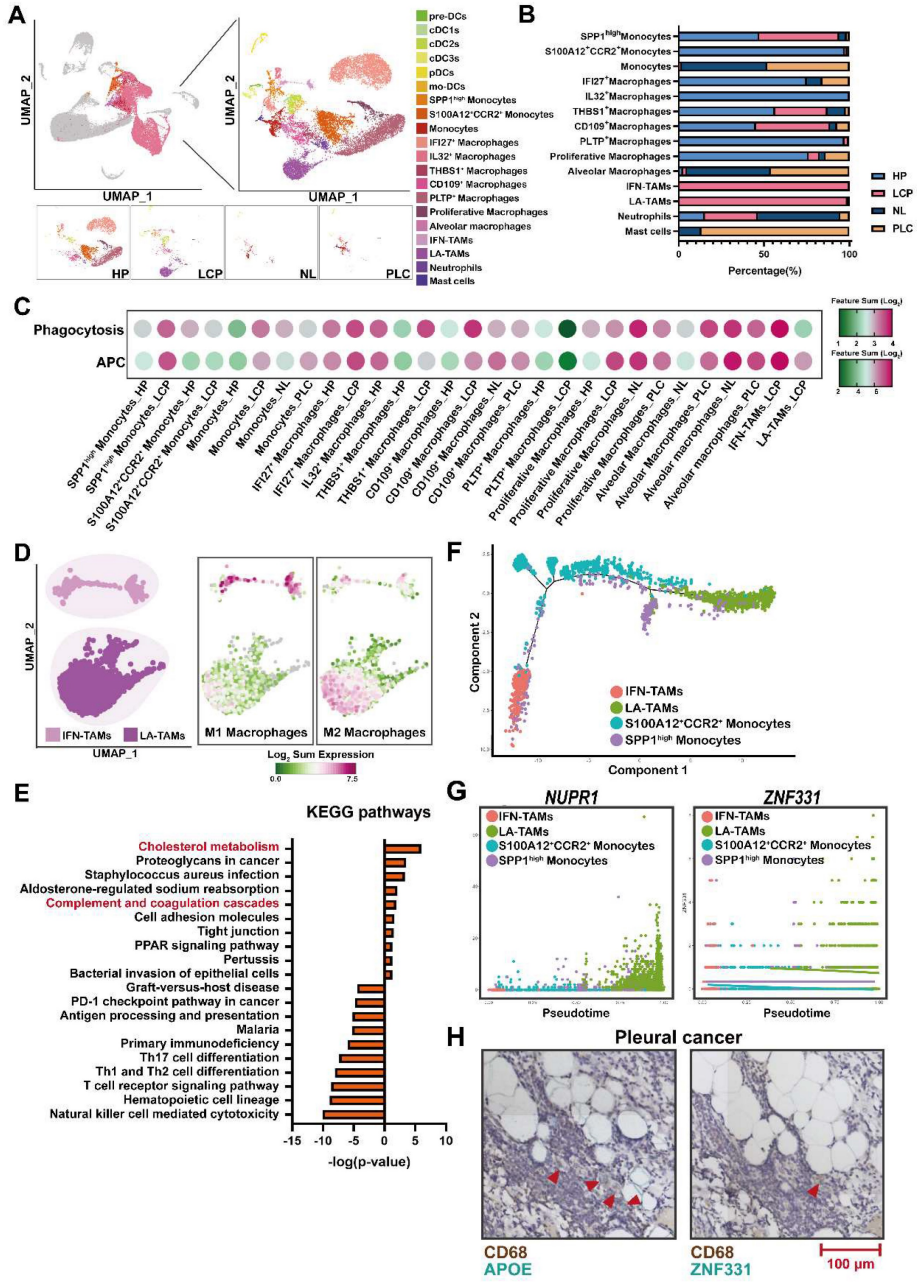
** Lipid-associated tumor-associated macrophages (LA-TAMs) exerted an immunosuppressive phenotype in the pleural microenvironment.** (A) UMAP plots of myeloid cells in the HP, LCP, NL, and PLC groups. (B) The percentage of different myeloid cell subsets. (C) The functional scores of monocytes/macrophages. (D) The phenotype of two TAM subsets. (E) KEGG pathway analysis of LA-TAMs. (F) Trajectory analysis of LA-TAMs. (G) Expressions of different transcriptional factors across monocytes and TAMs, ordered by Monocle 2 analysis in pseudotime. (H) CD68^+^APOE^+^ LA-TAMs presence and CD68^+^ZNF331^+^ LA-TAMs in pleural lung cancer. Arrowhead indicated LA-TAMs.

To characterize the subsets of myeloid cells, we re-clustered monocytes/macrophages/DCs. As shown in Figure 4A, 19 sub-populations with specific gene signatures, including 3 groups of monocytes, 10 groups of macrophages, and 6 groups of DCs were characterized (Figure 4A, Figure S5A, and Table S8). Of note, interferon-primed TAMs (IFN-TAMs; IFITM3^+^, CASP4^+^) and lipid-associated TAMs (LA-TAMs; APOE^+^, GPNMB^+^) subsets were preferentially enriched in the LCP group, while S100A12^+^CCR2^+^ monocytes, IFI27^+^ macrophages, and IL32^+^ macrophages were abundant in the HP group. In addition, PLTP^+^ macrophages and proliferative macrophages were largely decreased in the LCP group (Figure 4B). Functional score analysis revealed that IFN-TAMs and proliferative macrophages had higher expressions of antigen-presenting cells (APCs) and phagocytosis, whereas PLTP^+^ macrophages lost their APC function and phagocytosis in the LCP group compared with those in the pleural fluid of the HP group (Figure 4C and Table S9).

We then focused on two TAM subsets in the pleural fluid of the LCP group. Phenotypic analysis showed that IFN-TAMs exhibited the M1-like phenotype, whereas LA-TAMs exhibited M2-like type macrophages with lower expressions of APCs and phagocytosis capacity (Figure 4D and Figure S5B). Pathway analysis indicated that Cholesterol metabolism and Complement and coagulation cascades were associated with LA-TAMs, while IFN-priming was associated with IFN-TAMs (Figure 4E and Figure S5C-E). To understand the transcriptional transition from monocytes to the two types of TAMs, we performed an unsupervised trajectory analysis to assess the changes in the status of monocytes and macrophages presented in the LCP group (Figure 4F). The results showed that transcription factors/activators *NUPR1* and *ZNF331* were upregulated (Figure 4G). In addition, soluble factors including *HAMP*, *FN1*, and *A2M* were increased in the LA-TAMs (Figure S5F). IHC data showed the infiltrations of LA-TAMs (CD68^+^APOE^+^ and CD68^+^ZNF331^+^) were found in pleural lung cancers (Figure 4H).

### The level of complement component C5 was associated with overall survival of EGFR-mutated lung cancer with pleural metastasis

**Figure 5:**
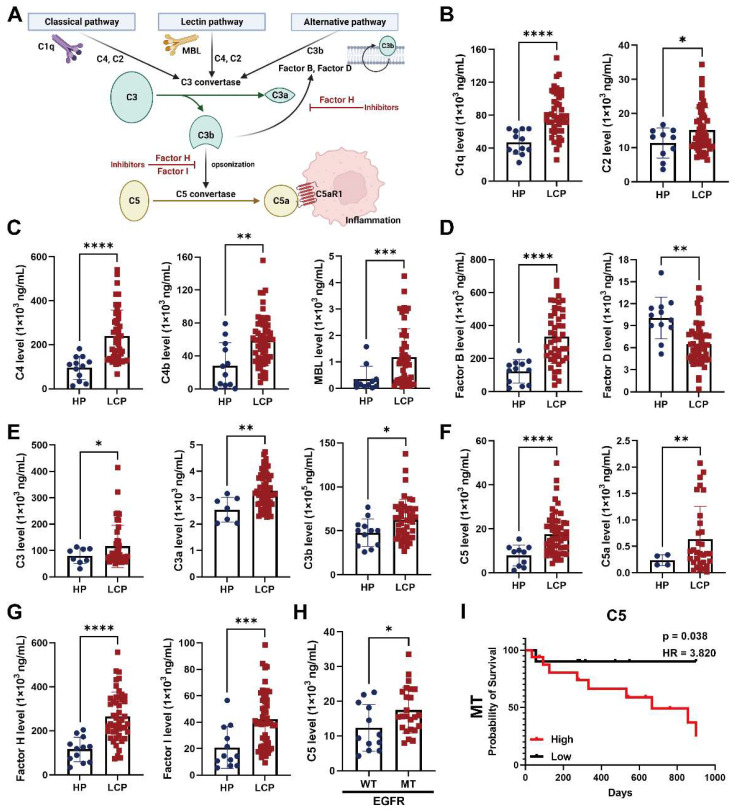
** The levels of complement factors in fluid of MPE.** (A) Scheme of the complement pathways. The concentrations of complement factors of (B) classical, (C) lectin pathway, and (D) alternative pathways. The levels of (E) C3 and (F) C5 -related factors. (G) The levels of complement factors H and I in pleural fluid. (H) The level of C5 in pleural fluid of patient with WT or MT EGFR. (I) C5 were associated with poor overall survival in lung cancer patients with MT EGFR. Various complement factors in pleural fluid of HP and LCP were determined by EMD Millipore's MILLIPLEX^®^ Complement Panel 1 and 2 Magnetic Bead Panel. HR, hazard ratio. Data are presented as mean ± SD. *p < 0.05, **p < 0.01, ***p < 0.001, ****p < 0.0001.

Since complement and coagulation cascades were associated with LA-TAMs, we assessed the roles of various complement components in pleural metastasis. The results showed that several complement components, including classical pathway factors (C1q, C2), lectin pathway factors (C4, C4b, MBL), alternative pathway factors (complement factor B), C3 and its cleavage product C3a and C3b, C5 and its cleavage product C5a, complement factor H, and complement factor I were increased in the pleural fluid of LCP group, while complement factor D was decreased compared with the HP group (Figure 5A-G). In addition to C1q, C4b, MBL, and complement factor B were significantly elevated in EGFR mutated pleural fluid, whereas the levels of other complement factors in the pleural fluid of LCP group were not significantly different between patients with wild type (WT) EGFR and mutated (MT) EGFR (Figure S6A-F). C5 was also significantly increased in MT EGFR, and Kaplan-Meier analysis showed that higher levels of C5 were associated with poorer overall survival in the LCP with MT EGFR, but not in those with WT EGFR (Figure 5H-I and Figure S7A). Other complement factors were not associated with the overall survival of the LCP regardless of EGFR mutation status (Figure S7B-L).

### Heterogeneity of the DC landscape of the pleural microenvironment

**Figure 6:**
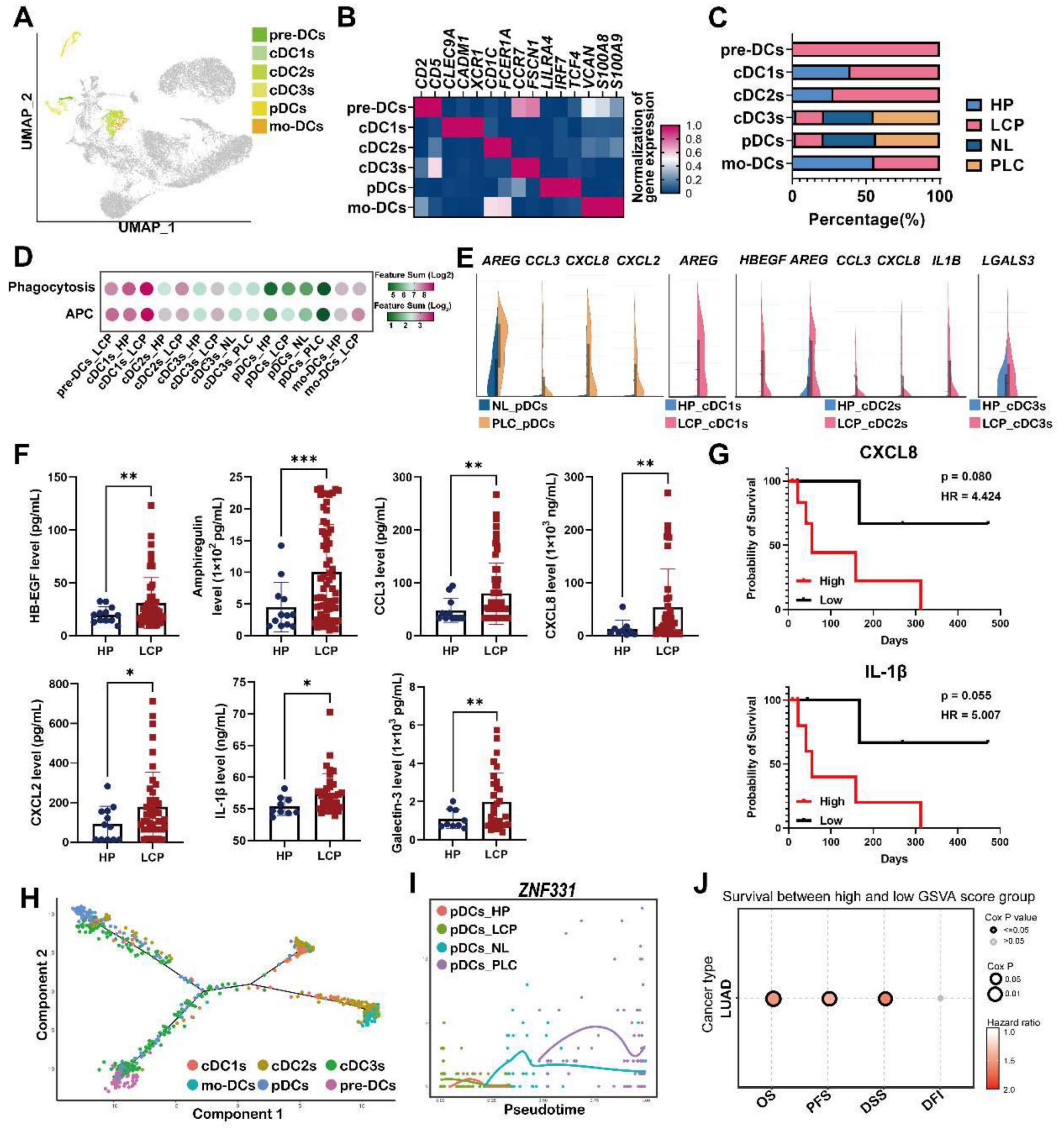
** Dysregulation of pDCs at the primary tumor site.** (A) Visualization of six clusters of DCs on the UMAP plot. (B) Cell markers used to annotate DC clusters. (C) Cell populations of various DC subsets. (D) Functional scores of the DC subsets. (E) Expressions of pro-tumorigenic factors in the DC subsets. (F) The levels of HB-EGF, amphiregulin, CCL3, CXCL2, CXCL8, and IL-1β in the pleural fluid. (G) The impact of CXCL8 and IL-1β in the overall survival of lung cancer patients. (H) Trajectory analysis of DCs in pleural fluid inferred by Monocle 2 software. (I) Expression of *ZNF331* across DC subsets. (J) The gene set of pDCs in PLC was associated with shorter overall survival in the lung cancer patients. Data are presented as mean ± SD. *p < 0.05, **p < 0.01, ***p < 0.001, MPE, malignant pleural effusion.

To investigate the increased number of cells and heterogeneity in functional subtypes of DCs in the LCP group, we performed unsupervised re-clustering on the DCs using a UMAP algorithm. The results yielded 4 subclusters, including precursor dendritic cells (pre-DCs), monocyte-derived DCs (mo-DCs), conventional DCs (cDC1s, cDC2s, and cDC3s), and plasmacytoid DCs (pDCs) based on cell markers, functional, and developmental differences (Figure 6A-B). Pre-DCs were rarely found in the HP group, but they were found in the LCP group. The number of mo-DCs slightly decreased, whereas the number of cDCs (cDC1-3) and pDCs increased in the LCP group compared with the HP group (Figure 6C). Functional changes of each type of DC were assessed by functional scores. cDC1 cluster exhibited the highest APC function among all DC subsets, however, there was no obvious change in APC ability in most DC subsets between the HP and LCP groups, except for cDC1 and mo-DC subsets of LCP. In contrast, cDC1 and mo-DC clusters exhibited the highest phagocytosis function among all DC subsets. The phagocytotic ability increased in all cDCs and mo-DCs in the LCP group. In contrast to the DCs in the pleura, pDCs exhibited noticeably reduced APC and phagocytosis scores in the PLC group compared with the pDCs in the NL group (Figure 6D).

Notably, pDCs expressed pro-tumorigenic factors, *AREG*, *CCL3*, *CXCL8*, and *CXCL2* in the PLC group, while cDC1 and cDC2 expressed *AREG, HBEGF*, *CCL3*, *CXCL8*, and *IL1B* in the LCP group compared with NL and HP, respectively. In addition, cDC3s expressed* LGALS3* in the LCP group compared with HP group (Figure 6E). ELISA also revealed a higher expression of these factors at protein levels in the LCP group compared with the HP group (Figure 6F). However, these factors were not associated with the overall survival of the LCP regardless of their EGFR mutation status (Figure S8A-C). Higher levels of CXCL8 and IL-1β have trends with poorer overall survival in the LCP with WT EGFR (Figure 6G). To investigate which hub genes contributed to the functional change of pDCs in the PLC group, we performed cell lineage trajectory analysis, which revealed that *ZNF331* was gradually upregulated along with the pDC differentiation trajectory in the PLC group (Figure 6H-I). We performed survival analysis of The Cancer Genome Atlas Lung Adenocarcinoma Cohort (TCGA LUAD) using the GSCA tool and confirmed that the pDC cluster gene sets (log_2_ FC > 3.5 and p < 0.05) were significantly associated with poor overall survival (OS, p = 0.0055), progression-free survival (PFS, p = 0.008) and disease specific survival (DSS, p = 0.021), making them a potentially useful indicator of adverse clinical outcomes in lung adenocarcinoma (Figure 6J).

### Cell-cell interactions in the pleural microenvironment

**Figure 7:**
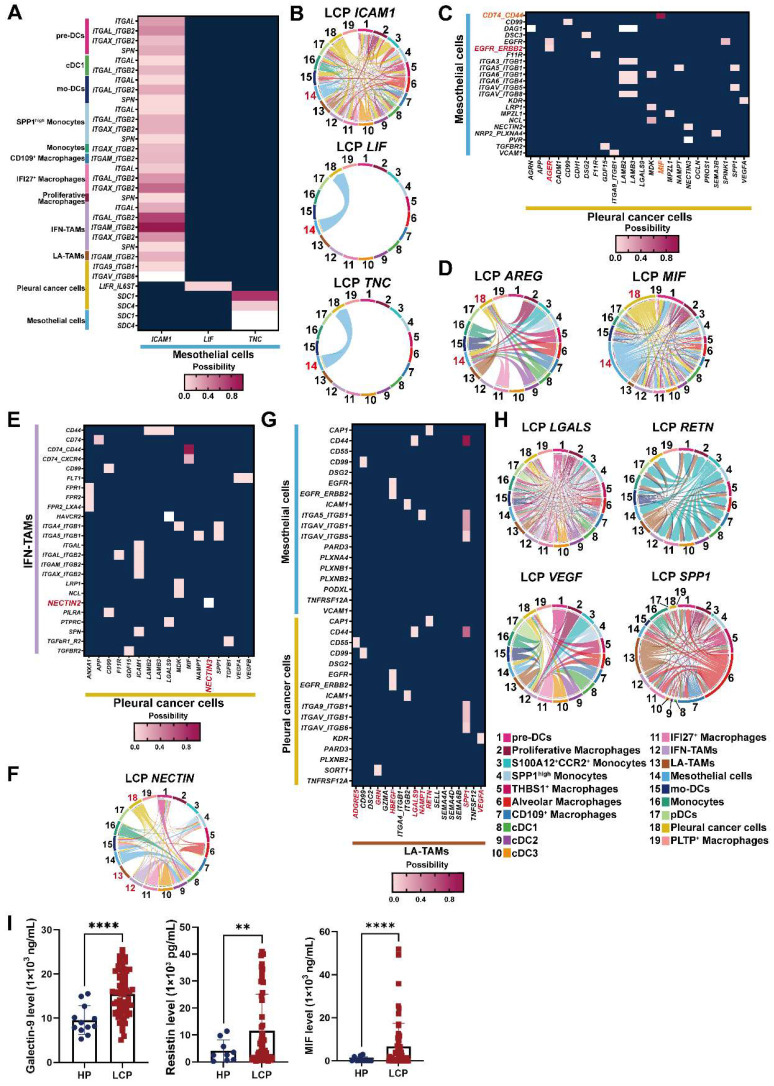
** Cross-talk of mesothelial cells, cancer cells, and LA-TAMs in the pleural niche.** (A) Heatmap showing the putative receptor-ligand interactions between mesothelial cells and immune cells. (B) Circular plot displayed the impact of mesothelial cells and cancer cells or DCs. Lines originated from the ligand and connected to its receptor as indicated by the arrowhead. (C) Interactions of pleural cancer cells with mesothelial cells. (D) Circular plot displayed the impact of pleural cancer cells on mesothelial cells. (E) Interactions of pleural cancer cells with IFN-TAMs. (F) Circular plot displays the impact of pleural cancer cells on immune cells. (G) Cell-cell interactions between LA-TAMs and cancer cells/mesothelial cells. (H) Circular plot of LA-TAMs and cancer/mesothelial cells axis. (I) Levels of galectin-9, resistin, and MIF in the pleural fluid. Data are presented as mean ± SD. **p < 0.01, ****p < 0.0001.

Differentially expressed gene (DEG) ligand-receptor pairs within cell subtypes of the pleural ecosystem allowed us to investigate potential communication between cell compartments that may help characterize the mechanism of pleural metastasis. Network analysis identified a key role of mesothelial cells, which communicated with cancer cells by *TNC* and *LIF*, and distinct immune cells, including all subtypes of DCs, macrophages, and monocytes by *ICAM1* (Figure 7A-B). Pleural cancer cells interacted with mesothelial cells by *MIF/CD74-CD44* complex and *AREG/EGFR-ERBB2* (Figure 7C-D and Figure S9A). Interestingly, pleural cancer cells communicated with IFN-TAMs through interactions with *NECTIN3/NECTIN2*, which was not found in LA-TAMs, suggesting this cross-talk was associated with anti-tumorigenic immunity (Figure 7E-F and Figure S9B). Pleural cancer cells also interacted with cDCs by *MIF* (Figure S9C). Similar to the potential cross-talk between mesothelial cells and cancer cells, we identified possible interactions between LA-TAMs/mesothelial cells by *SPP1*, *RETN*, *LGALS9*, *NAMPT*, and *HBEGF* secretion. LA-TAMs also participated in the modification of cancer cells by *SPP1*, *VEGFA*, *RETN*, *GRN, ADGRE5*, and *HBEGF* secretion (Figure 7G-H, Figure S9D and Table S10).

To confirm the interactions of cell subsets in the pleural ecosystem, we measured the factors involved in cell-cell communication. The results showed that MIF and galectin-9 (*LGALS9*) were higher in the pleural fluid of the LCP group than in the HP group (Figure 7I). However, these factors were not associated with the overall survival of the LCPs regardless of EGFR mutation status (Figure S9E-G).

## Discussion

Malignant pleural effusion is a common but complicated situation leading to impaired lung function and worse quality of life 17. However, the pleural microenvironment during cancer metastasis remains largely unknown. In the current study, we demonstrated the important contributions of PMCs and LA-TAMs in pleural metastasis, and we also elucidated the mechanisms underlying the survival of metastatic cancer in pleural fluid. Our results revealed that mesothelial cells were reprogrammed to the mesenchymal phenotype, as characterized by the upregulation of ECM proteins. The anti-ferroptosis ability of cancer cells may prevent death when they suffer from stress in the pleural cavity. We also established mechanistic alterations in the formation of a pleural ecosystem for lung cancer metastasis.

PMCs have been shown to change their phenotype from an epithelial phenotype to a profibrotic phenotype in response to various soluble factors during pathogenesis, such as inflammation 18, 19. During phenotypic conversion, a process known as mesothelial to mesenchymal transition (MesoMT), mesothelial cells express α-smooth muscle actin (α-SMA) and vimentin, and secrete ECM proteins such as collagen I and fibronectin 19. Furthermore, mesothelial cells with upregulated expressions of *ICAM1* and *VEGF* have been observed in the pleural cavity contributing to angiogenesis and MPE formation 20, 21. Most functional studies have focused on the mechanisms of peritoneal dissemination in cancers. Increasing evidence has shown that peritoneal mesothelial cells undergo MesoMT during peritoneal dissemination in colon, gastric, ovarian, and pancreatic cancers 22, 23. Lung adenocarcinoma-derived extracellular vesicles containing miR-21 have been reported to induce MesoMT in pre-metastatic niches of dissemination in the pleural cavity 24. In this study, compared with the control ones, patients with lung adenocarcinoma showed notably increased expression of MesoMT markers, including N-cadherin (*CDH2*), α-SMA (*ACTA2*), and vimentin (*VIM*) in PMCs, which overlapped with those of MesoMT transcription factors (*SNAI1/2* and *ZEB1/2*) to a great extent. In addition, we evaluated the ECM components, tenascin C (*TNC*), fibronectin (*FN1*), ICAM-1 (*ICAM1*), as well as VEGFA in the mesothelial cells of the LCP. Higher levels of Tenascin C (*TNC*), ICAM1, and VEGFA further supported that they were involved in pleural metastasis in lung cancer. *In vitro* experiments revealed that lung cancer cells (A549, CL1-5, H1975, and HCC827) stimulated mesothelial cells (MeT-5A) to undergo MesoMT. Taken together, these data offer the proof of concept that alterations of mesothelial cells contribute to the pathogenesis of this devastating disease, pleural metastasis, possibly through modulating MesoMT.

The cellular and molecular mechanisms of pleural-specific metastasis in cancer are still mostly unclear 25. ECM detachment has been shown to cause a remarkable increase in reactive oxygen species, resulting in the ferroptosis of cancer cells during metastasis 26, 27. Recent studies have provided evidence that the intrinsic hypersensitivity of cancer cells to ferroptosis drives lymphatic metastasis 28. Pretreating melanoma with chemical inhibitors for ferroptosis resulted in greater metastasis through blood but not lymphatic system in an animal model, suggesting that the regulation of ferroptosis may determine the metastatic route 29.

Previous studies have indicated that oxidized polyunsaturated fatty acids, which can induce ferroptosis, were upregulated in MPE 30, 31, indicating that ferroptosis resistance is required for pleural metastasis. In our study, we identified ferroptosis resistance through the increase of ferroptosis suppressors (*NUPR1* and its target *GPX4* and *FTL*) and attenuation of the ferroptosis inducer, *ACSL4*, in the patients with pleural metastatic lung cancer. MPE as condition medium increased the expressions of *NUPR1*, *GPX4*, and *FTL* in A549 and H1975 cells. Moreover, inhibition of *GPX4* impaired the cell viability of GPX4 high-expressing A549 and H1975 cells cultured in the fluid of MPE, whereas overexpression of GPX4 rescued cell death from MPE in GPX4 low-expressing HCC827 cells, supporting the theory that ferroptosis resistance may be an essential factor related to pleural metastasis in lung cancer.

Defective antigen presentation represents a major immune escape mechanism and also contributes to immunotherapy failure in malignancy 32. Innate immune cells, including macrophages and DCs, are the major APCs as the first line defense against exotic organisms by up-taking exogenous antigens 33. Novel subset-specific markers have recently been identified, including monocyte-derived STAB1^+^TREM2^high^ LA-TAMs in gastric, colorectal, lung, and breast cancer 34, 35. LA-TAMs have been proposed to inhibit antitumor immunity and may promote tumor progression, because lipid catabolism is associated with immunosuppression and immune tolerance-related functions, while lipid anabolism is associated with inflammation in macrophages 36, 37. In this study, we found that LA-TAMs expressed a suppressive M2 phenotype and reduced phagocytosis as well as APC function in the pleural niche for cancer metastasis, while pDCs lost their function at the primary cancer site. In addition, both of them expressed high levels of pro-tumorigenic factors, including several ligands for EGFR and inflammatory cytokines/chemokines (CCL3, CXCL2, and CXCL8). The gene set determining the phenotype of pDCs also conferred poor clinical outcomes in the primary lung cancer patients. Therefore, based on their changes in phenotype and function, LA-TAMs and pDCs contributed to a unique pleural and lung immune ecosystem, aiding the construction of an immunosuppressive and pro-tumorigenic niche favorable for lung cancer.

*ZNF331* (zinc-finger protein 331) belongs to the zinc-finger gene family, encoding a zinc finger protein which contains a Krüppel-associated box domain that plays an essential role in the transcriptional regulation process. Aberrant promoter hypermethylation of *ZNF331* was proved to be associated with cancers 38, 39. In contrast, a recent study demonstrated that high *ZNF331* methylation in peripheral blood leukocytes significantly decreased the risk of gastric cancer 40, showing that *ZNF331* is also involved in regulation of the immune system. Our results showed that *ZNF331* was involved in the transition of both LA-TAMs and pDCs, which exhibited a dysregulated immune phenotype, suggesting it may regulate the anti-cancer function of myeloid cells. However, research on *ZNF331* in other immune system is scarce and further studies are necessary to elucidate this issue.

As a modulator and effector of innate immune responses, complement activation not only provides protection against pathogens, but also participates in tumorigenesis 41. Complement activation has been implicated as a driver of tumor growth and metastasis in the tumor microenvironment. Blockade of the C3 or C5a‐C5AR1 axis was shown to impair cancer growth and bone metastasis of lung cancer in an animal model 42, 43. In the present study, we found that the levels of several complement factors involved in classical, lectin, and alternative pathways were enhanced in MPE. High levels of C5 in MPE were significantly associated with shorter overall survival. Disruption of C5 signaling may abrogate their tumor-associated osteoclastogenic activity, thereby impairing osseous colonization 42. Thus, to effectively prevent and treat pleural metastasis, therapeutic strategies which block C5-related signaling could be considered to improve the unique immunological characteristics in the pleural ecosystem in lung cancer patients with EGFR mutation. Further studies should explore the specific ecosystem characteristics of the pleura in lung cancer patients with EGFR mutation by comparing the pleural metastatic niche between those with wild type and mutated EGFR.

Taken together, our findings provide a high-resolution single-cell atlas of the pleural niche during cancer metastasis. This atlas identified previously unrecognized changes in gene expressions and cellular interactions in primary cancer and metastatic cancer, mesothelial cells and myeloid immune cells, highlighting the complexity and diversity of cellular transitions and immunity in pleural metastasis of lung cancer. Future research validating the consequences of the specific transcriptional dysregulation investigated in this study may provide insights into the molecular mechanisms and potentially lead to the development of novel diagnostic strategies and personalized treatments for patients with lung cancer.

## Supplementary Material

Supplementary figures.

Supplementary tables.

## Abbreviations

A2M: alpha-2-macroglobulin
ACSL4: acyl-CoA synthetase long chain family member 4
ACTA2: actin alpha 2
ADGRE5: adhesion G protein-coupled receptor E5
ANOVA: analysis of variance
APC: antigen-presenting cell
APOE: apolipoprotein E
AREG: amphiregulin
α-SMA: alpha-smooth muscle actin
AT2: alveolar type II epithelial cell
ATCC: American Type Culture Collection
C1q: complement C1q
C2: complement C2
C3: complement C3
C3a: complement C3a
C3b: complement C3b
C4: complement C4
C4b: complement C4b
C5: complement C5
C5a: complement C5a
C5AR1: complement protein coding C5a receptor 1
CALB1: calbindin 1
CASP4: caspase 4
CCL3: C-C motif chemokine ligand 3
CCR2: C-C motif chemokine receptor 2
CD44: cluster of differentiation (CD)-44
CD68: cluster of differentiation (CD)-68
CD74: cluster of differentiation (CD)-74
cDC: conventional dendritic cell
CDH2: N-cadherin
cDNA: complementary DNA
CLDN1: claudin 1
CXCL2: C-X-C motif chemokine ligand 2
CXCL8: C-X-C motif chemokine ligand 8
DAB: 3,3'-Diaminobenzidine
DC: dendritic cell
DEG: differentially expressed gene
DDS: disease-specific survival
ECM: extracellular matrix
ECM1: extracellular matrix protein 1
EGFR: epidermal growth factor receptor
ELISA: enzyme-linked immunosorbent assay
ERBB2: Erb-B2 receptor tyrosine kinase 2
EREG: epiregulin
FBS: fetal bovine serum
FFPE: formalin-fixed paraffin-embedded
FN1: fibronectin 1
FTL: ferritin light chain
GAPDH: glyceraldehyde-3-phosphate dehydrogenase
GPNMB: glycoprotein nmb
GPX4: glutathione peroxidase 4
GRN: granulin precursor
GSCA: Gene Set Cancer Analysis
HAMP: hepcidin antimicrobial peptide
HB-EGF: heparin binding EGF like growth factor
HEPES: 4-(2-hydroxyethyl)-1-piperazineethanesulfonic acid
HP: pleural effusion with heart failure
HR: hazard ratio
HRP: horseradish peroxidase
ICAM1: intercellular adhesion molecule 1
IFI27: interferon alpha inducible protein 27
IFITM3: interferon induced transmembrane protein 3
IFN-TAM: interferon-primed tumor-associated macrophage
IHC: immunohistochemistry
IL-1β/IL1B: interleukin 1 beta
IL32: interleukin 32
IPA: Ingenuity Pathway Analysis
LA-TAM: lipid-associated tumor-associated macrophage
LCP: pleural effusion with lung cancer
LGALS3: galectin 3
LGALS9: galectin 9
LIF: LIF interleukin 6 family cytokine
MBL: mannose binding lectin
MesoMT: mesothelial-mesenchymal transition
MIF: macrophage migration inhibitory factor
MMP-2: matrix metallopeptidase 2
mo-DC: monocyte-derived dendritic cell
MPE: malignant pleural effusion
MT: mutated
MXI1: MAX interactor 1, dimerization protein
NAMPT: nicotinamide phosphoribosyltransferase
NECTIN2: nectin cell adhesion molecule 2
NECTIN3: nectin cell adhesion molecule 3
NK cell: natural killer cell
NL: normal lung
NUPR1: nuclear protein 1, transcriptional regulator
OS: overall survival
PAI-1: Plasminogen activator inhibitor-1
pDC: plasmacytoid dendritic cell
PFS: progression free survival
PLC: primary lung cancer
PLTP: phospholipid transfer protein
PMC: pleural mesothelial cell
pre-DC: precursor dendritic cell
qRT-PCR: quantitative real-time polymerase chain reaction
RETN: resistin
S100A12: S100 Calcium binding protein A12
scRNA-seq: single-cell RNA sequencing
SD: standard deviation
SERPINE1: serpin family E member 1
siRNA: small interfering RNA
SLC2A1: solute carrier family 2 member 1
SNAI1: Snail
SNAI2: Slug
SPP1: secreted phosphoprotein 1
STAB1: stabilin 1
TAM: tumor-associated macrophage
TCGA STAD: The Cancer Genome Atlas Stomach Adenocarcinoma Cohort
THBS-1: thrombospondin 1
TJP1: tight junction protein 1
TJP2: tight junction protein 2
TNC: tenascin C
TREM2: triggering receptor expressed on myeloid cells 2
UMAP: uniform manifold approximation and projection
UPK3B: uroplakin 3B
VEGFA: vascular endothelial growth factor A
VEGFB: vascular endothelial growth factor B
VIM: vimentin
WT: wild type
WT1: WT1 transcription factor
ZEB1: Zinc finger E-Box binding homeobox 1
ZEB2: Zinc finger E-Box binding homeobox 2
ZNF331: Zinc finger protein 331

## References

[1] Sung H, Ferlay J, Siegel RL, Laversanne M, Soerjomataram I, Jemal A (2021). Global cancer statistics 2020: GLOBOCAN estimates of incidence and mortality worldwide for 36 cancers in 185 countries. CA Cancer J Clin.

[2] Zhao Y, Yu L, Wang L, Wu Y, Chen H, Wang Q (2023). Current status of and progress in the treatment of malignant pleural effusion of lung cancer. Front Oncol.

[3] Koegelenberg CFN, Shaw JA, Irusen EM, Lee YCG (2018). Contemporary best practice in the management of malignant pleural effusion. Ther Adv Respir Dis.

[4] Psallidas I, Kalomenidis I, Porcel JM, Robinson BW, Stathopoulos GT (2016). Malignant pleural effusion: from bench to bedside. Eur Respir Rev.

[5] Terra RM, Dela Vega AJM (2018). Treatment of malignant pleural effusion. J Vis Surg.

[6] Karpathiou G, Benli J, Désage AL, Jacob M, Tiffet O, Peoc'h M (2022). Prognostic role of immune microenvironment in pleural metastases from breast and lung adenocarcinomas. Ann Transl Med.

[7] Tissot C, Gay P, Brun C, Froudarakis ME (2019). Novel insights into the systemic treatment of lung cancer malignant pleural effusion. Clin Respir J.

[8] Antony VB (2003). Immunological mechanisms in pleural disease. Eur Respir J.

[9] Li Q, Hu C, Su S, Ma Z, Geng Y, Hu Y (2022). Non-small cell lung cancer with malignant pleural effusion may require primary tumor radiotherapy in addition to drug treatment. Cancer Manag Res.

[10] Porcel JM, Gasol A, Bielsa S, Civit C, Light RW, Salud A (2015). Clinical features and survival of lung cancer patients with pleural effusions. Respirology.

[11] Agalioti T, Giannou AD, Stathopoulos GT (2015). Pleural involvement in lung cancer. J Thorac Dis.

[12] Wilson RB, Solass W, Archid R, Weinreich FJ, Königsrainer A, Reymond MA (2019). Resistance to anoikis in transcoelomic shedding: the role of glycolytic enzymes. Pleura Peritoneum.

[13] Conner JR, Cibas ES, Hornick JL, Qian X (2014). Wilms tumor 1/cytokeratin dual-color immunostaining reveals distinctive staining patterns in metastatic melanoma, metastatic carcinoma, and mesothelial cells in pleural fluids: an effective first-line test for the workup of malignant effusions. Cancer Cytopathol.

[14] Zhang Z, Ji W, Huang J, Zhang Y, Zhou Y, Zhang J (2022). Characterization of the tumour microenvironment phenotypes in malignant tissues and pleural effusion from advanced osteoblastic osteosarcoma patients. Clin Transl Med.

[15] Jin S, Guerrero-Juarez CF, Zhang L, Chang I, Ramos R, Kuan C-H (2021). Inference and analysis of cell-cell communication using CellChat. Nat Commun.

[16] Gao L, Nie X, Gou R, Hu Y, Dong H, Li X (2021). Exosomal ANXA2 derived from ovarian cancer cells regulates epithelial-mesenchymal plasticity of human peritoneal mesothelial cells. J Cell Mol Med.

[17] Jany B, Welte T (2019). Pleural effusion in adults-etiology, diagnosis, and treatment. Dtsch Arztebl Int.

[18] Sakai T, Choo YY, Sato O, Ikebe R, Jeffers A, Idell S (2022). Myo5b transports fibronectin-containing vesicles and facilitates FN1 secretion from human pleural mesothelial Cells. Int J Mol Sci.

[19] Qian G, Adeyanju O, Roy S, Sunil C, Jeffers A, Guo X (2022). DOCK2 Promotes pleural fibrosis by modulating mesothelial to mesenchymal transition. Am J Respir Cell Mol Biol.

[20] Lieser EA, Croghan GA, Nevala WK, Bradshaw MJ, Markovic SN, Mansfield AS (2013). Up-regulation of pro-angiogenic factors and establishment of tolerance in malignant pleural effusions. Lung cancer.

[21] Zhao J, Liu B, Liu N, Zhang B, He X, Ma Q (2022). The role of angiogenesis in malignant pleural effusion: from basic research to clinical application. Am J Cancer Res.

[22] Gordillo CH, Sandoval P, Muñoz-Hernández P, Pascual-Antón L, López-Cabrera M, Jiménez-Heffernan JA (2020). Mesothelial-to-mesenchymal transition contributes to the generation of carcinoma-associated fibroblasts in locally advanced primary colorectal carcinomas. Cancers.

[23] Pascual-Antón L, Cardeñes B, Sainz de la Cuesta R, González-Cortijo L, López-Cabrera M, Cabañas C (2021). Mesothelial-to-mesenchymal transition and exosomes in peritoneal metastasis of ovarian cancer. Int J Mol Sci.

[24] Watabe S, Kikuchi Y, Morita S, Komura D, Numakura S, Kumagai-Togashi A (2020). Clinicopathological significance of microRNA-21 in extracellular vesicles of pleural lavage fluid of lung adenocarcinoma and its functions inducing the mesothelial to mesenchymal transition. Cancer Med.

[25] Ruan X, Sun Y, Wang W, Ye J, Zhang D, Gong Z (2020). Multiplexed molecular profiling of lung cancer with malignant pleural effusion using next generation sequencing in Chinese patients. Oncol Lett.

[26] Qiu L, Zhou R, Zhou L, Yang S, Wu J (2022). CAPRIN2 upregulation by LINC00941 promotes nasopharyngeal carcinoma ferroptosis resistance and metastatic colonization through HMGCR. Front Oncol.

[27] Brown CW, Amante JJ, Goel HL, Mercurio AM (2017). The α6β4 integrin promotes resistance to ferroptosis. J Cell Biol.

[28] Xie X, Tian L, Zhao Y, Liu F, Dai S, Gu X (2023). BACH1-induced ferroptosis drives lymphatic metastasis by repressing the biosynthesis of monounsaturated fatty acids. Cell Death Dis.

[29] Ubellacker JM, Tasdogan A, Ramesh V, Shen B, Mitchell EC, Martin-Sandoval MS (2020). Lymph protects metastasizing melanoma cells from ferroptosis. Nature.

[30] Yang Z, Song Z, Chen Z, Guo Z, Jin H, Ding C (2020). Metabolic and lipidomic characterization of malignant pleural effusion in human lung cancer. J Pharm Biomed Anal.

[31] Shi Z, Zhang L, Zheng J, Sun H, Shao C (2021). Ferroptosis: biochemistry and biology in cancers. Front Oncol.

[32] Burrack AL, Schmiechen ZC, Patterson MT, Miller EA, Spartz EJ, Rollins MR (2022). Distinct myeloid antigen-presenting cells dictate differential fates of tumor-specific CD8^+^ T cells in pancreatic cancer. JCI insight.

[33] Park J, Wang L, Ho P-C (2022). Metabolic guidance and stress in tumors modulate antigen-presenting cells. Oncogenesis.

[34] Timperi E, Gueguen P, Molgora M, Magagna I, Kieffer Y, Lopez-Lastra S (2022). Lipid-associated macrophages are induced by cancer-associated fibroblasts and mediate immune suppression in breast cancer. Cancer Res.

[35] Ma RY, Black A, Qian BZ (2022). Macrophage diversity in cancer revisited in the era of single-cell omics. Trends Immunol.

[36] Huggins DN, LaRue RS, Wang Y, Knutson TP, Xu Y, Williams JW (2021). Characterizing macrophage diversity in metastasis-bearing lungs reveals a lipid-associated macrophage subset. Cancer Res.

[37] Jaitin DA, Adlung L, Thaiss CA, Weiner A, Li B, Descamps H (2019). Lipid-associated macrophages control metabolic homeostasis in a Trem2-dependent manner. Cell.

[38] Yu J, Liang QY, Wang J, Cheng Y, Wang S, Poon TCW (2013). Zinc-finger protein 331, a novel putative tumor suppressor, suppresses growth and invasiveness of gastric cancer. Oncogene.

[39] Wang Y, He T, Herman JG, Linghu E, Yang Y, Fuks F (2017). Methylation of ZNF331 is an independent prognostic marker of colorectal cancer and promotes colorectal cancer growth. Clin Epigenetics.

[40] Nie C, Han X, Wei R, Leonteva A, Hong J, Du X (2021). Association of ZNF331 and WIF1 methylation in peripheral blood leukocytes with the risk and prognosis of gastric cancer. BMC cancer.

[41] Lawal B, Tseng SH, Olugbodi JO, Iamsaard S, Ilesanmi OB, Mahmoud MH (2021). Pan-cancer analysis of immune complement signature C3/C5/C3AR1/C5AR1 in association with tumor immune evasion and therapy resistance. Cancers.

[42] Ajona D, Zandueta C, Corrales L, Moreno H, Pajares MJ, Ortiz-Espinosa S (2018). Blockade of the complement C5a/C5aR1 axis impairs lung cancer bone metastasis by CXCL16-mediated effects. Am J Respir Crit Care Med.

[43] Kleczko EK, Poczobutt JM, Navarro AC, Laskowski J, Johnson AM, Korpela SP (2022). Upregulation of complement proteins in lung cancer cells mediates tumor progression. Front Oncol.

