# Supplementary material for: Characterization of the pleural microenvironment niche and cancer transition using single-cell RNA sequencing in EGFR-mutated lung cancer
Source: Theranostics. 2023 Aug 6;13(13):4412–29. doi: 10.7150/thno.85084 (PMC10465223; doi:10.7150/thno.85084)
Supplement: Supplementary file 1 — Supplementary figures. [file thnov13p4412s1.pdf]

Supplementary data

Figure S1. Quality control of the single-cell NGS (A) The UMI number and percentage of the mitochondria. (B) The cell number in the normal lung (NL), primary lung cancer (PLC), pleural fluid of congestive heart failure (HP), and pleural metastasis (LCP) groups.

Figure S2. The cell profiles of primary and pleural metastasis tissue as determined by scRNA-seq. (A) Co-expressions of various cell markers on different cell clusters on the UMAP plot. (B) Proportion of various cell types. (C) Percentage of different cell types in different patients.

Figure S3. The MesoMT of pleural mesothelial cells (PMCs). (A) Visualization of PMCs by different patient groups. (B) Upregulated *ICAM1*, *SLC2A1,* and *TNC* in LCP. (C) The levels of MMP2 in pleural fluid obtained from the patients with congestive heart failure or pleural metastasis. (D) No significant difference of various factors in the pleural fluid of pleural metastasis lung cancer patients with wild type (WT) between mutated (MT) EGFR status. The impact of various secretory factors on the overall survival of lung cancer patients with WT (E) or MT (F) EGFR. Data are presented as mean ± SD. ns, not significant.

Figure S4. The evolution of cancer cells in the pleural niche. (A) InferCNV analysis of epithelial cells v.s. primary lung cancer and mesothelial cells v.s. pleural metastatic cancer. (B) The volcano plot of DEGs. Wiki pathway analysis (C) and IPA (D) of DEGs. (E) Heatmap of the ferroptosis driver gene set in pleural cancer. (F) Weight analysis of the ferroptosis driver gene set. (G) IHC showed upregulated GPX4 in pleural tumor than that in primary tumor in a paired tissue section. (H) The knockdown efficacy of *GPX4* siRNA. (I) Inhibition of *GPX4* by siRNA decreased cell viability of A549 in MPE. (J) A GPX4 inhibitor reduced cell viability of A549 in MPE. The cells were transfected with control or *GPX4* siRNA and the expression of GPX4 were assessed

after 24 h transfection by qRT-PCR. The data were presented as mean ± SD. ***$p < 0.001$.

Figure S5. LA-TAMs exhibited an immunosuppressive phenotype. (A) Cell markers of myeloid cells. Heatmaps of M1/M2 phenotype (B), complement (C), cholesterol metabolism (D), and IFN-related (E) pathways in IFN-TAMs and LA-TAMs. (F) The expressions of secretory factors across the monocytes and TAMs assessed by trajectory analysis.

Figure S6. The levels of complement factors in MPE of lung cancer patients with wild-type (WT) or mutated (MT) EGFR. The concentrations of complement factors of (A) classical pathway, (B) lectin pathway, (C) alternative pathway, (D) C3 and (E) C5-related factors, and (F) complement factor H and complement factors I in the pleural fluid of pleural metastatic lung cancer patients with WT or MT EGFR. ns, not significant. *$p < 0.05$, **$p < 0.01$.

Figure S7. Kaplan-Meier curves representing overall survival (OS) in the cohort stratified by complement factor expressions. The impact of complement factors of (A) C5-related factors, (B) classical pathway, (C) lectin pathway, (D) alternative pathway, (E) C3-related, and (F) complement factors H and I on the OS of pleural metastatic lung cancer patients with WT EGFR. The impact of complement factors of (G) C5a, (H) classical pathway, (I) lectin pathway, (J) alternative pathway, (K) C3-related, and (L) complement factors H and I in the OS of pleural metastatic lung cancer patients with MT EGFR. The OS of patients were calculated using the Kaplan-Meier method. CFB, complement factor B. CFD, complement factor D. CFH, complement factor H. CFI, complement factor I.

Figure S8. The levels of DC-derived factors in the pleural fluid of MPE. (A) The levels of various factors in the pleural fluid of MPE obtained from lung cancer patients with WT or MT EGFR. The impact of various DC-derived factors on the OS of WT

EGFR (B) and MT (C) lung cancer patients. ns, not significant.

Figure S9. The levels of various ligands involved in the cell-cell interaction of pleural microenvironment. (A) The interaction of pleural cancer cells by autocrine pattern. (B) The interaction of pleural cancer cells with LA-TAMs. (C) The interaction of pleural cancer cells with the subsets of DCs. (D) The interaction of IFN-TAMs with pleural cancer cells or mesothelial cell. (E) The levels of various factors in the MPE obtained from lung cancer patients with WT or MT EGFR. The impact of various ligands on the OS of EGFR wild type (F) and mutated (G) lung cancer patients. ns, not significant.

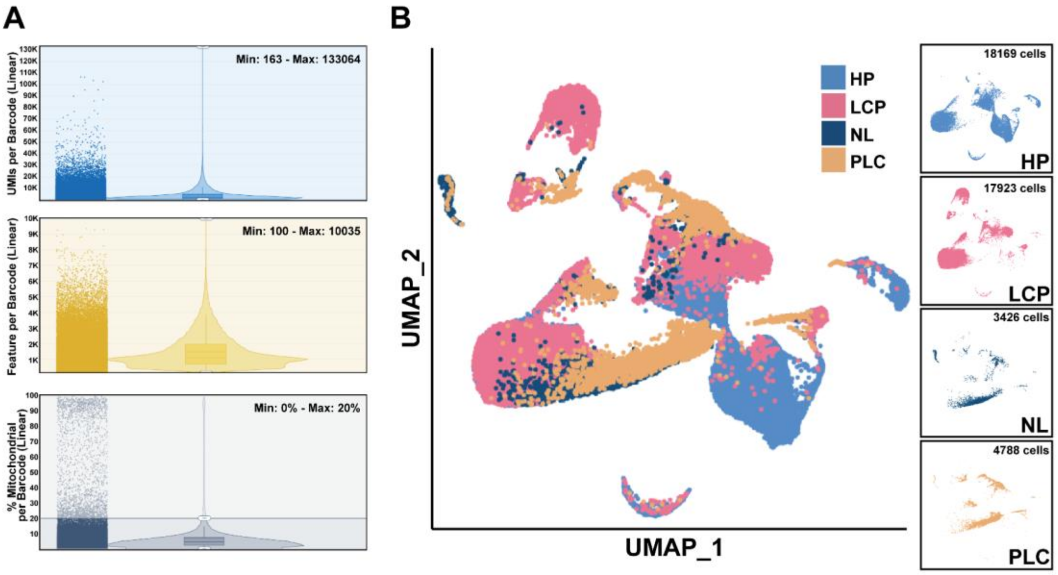
A
Min: 163 - Max: 133064
UMIs per Barcode (Linear)
Min: 100 - Max: 10035
Feature per Barcode (Linear)
Min: 0% - Max: 20%
% Mitochondrial per Barcode (Linear)
B
HP
LCP
NL
PLC
UMAP_1
UMAP_2
18169 cells
17923 cells
3426 cells
4788 cells

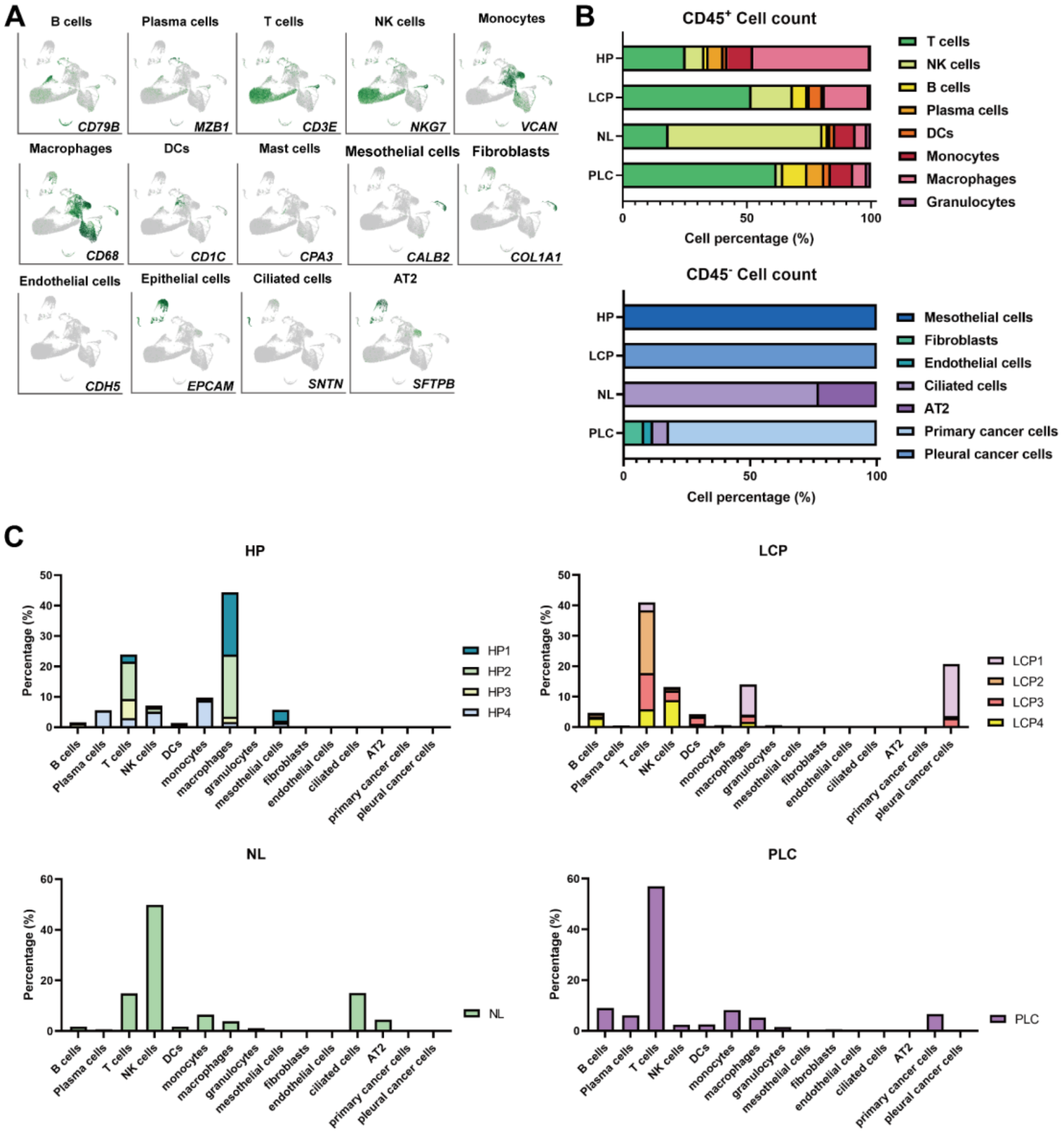
A
B cells
Plasma cells
T cells
NK cells
Monocytes
CD79B
MZB1
CD3E
NKG7
VCAN
Macrophages
DCs
Mast cells
Mesothelial cells
Fibroblasts
CD68
CD1C
CPA3
CALB2
COL1A1
Endothelial cells
Epithelial cells
Ciliated cells
AT2
CDH5
EPCAM
SNTN
SFTPB
B
CD45+ Cell count
HP
LCP
NL
PLC
0
50
100
Cell percentage (%)
T cells
NK cells
B cells
Plasma cells
DCs
Monocytes
Macrophages
Granulocytes
CD45- Cell count
Mesothelial cells
Fibroblasts
Endothelial cells
Ciliated cells
AT2
Primary cancer cells
Pleural cancer cells
C
HP
LCP
NL
PLC
Percentage (%)
HP1
HP2
HP3
HP4
LCP1
LCP2
LCP3
LCP4
B cells
Plasma cells
T cells
NK cells
DCs
monocytes
macrophages
granulocytes
mesothelial cells
fibroblasts
endothelial cells
ciliated cells
AT2
primary cancer cells
pleural cancer cells

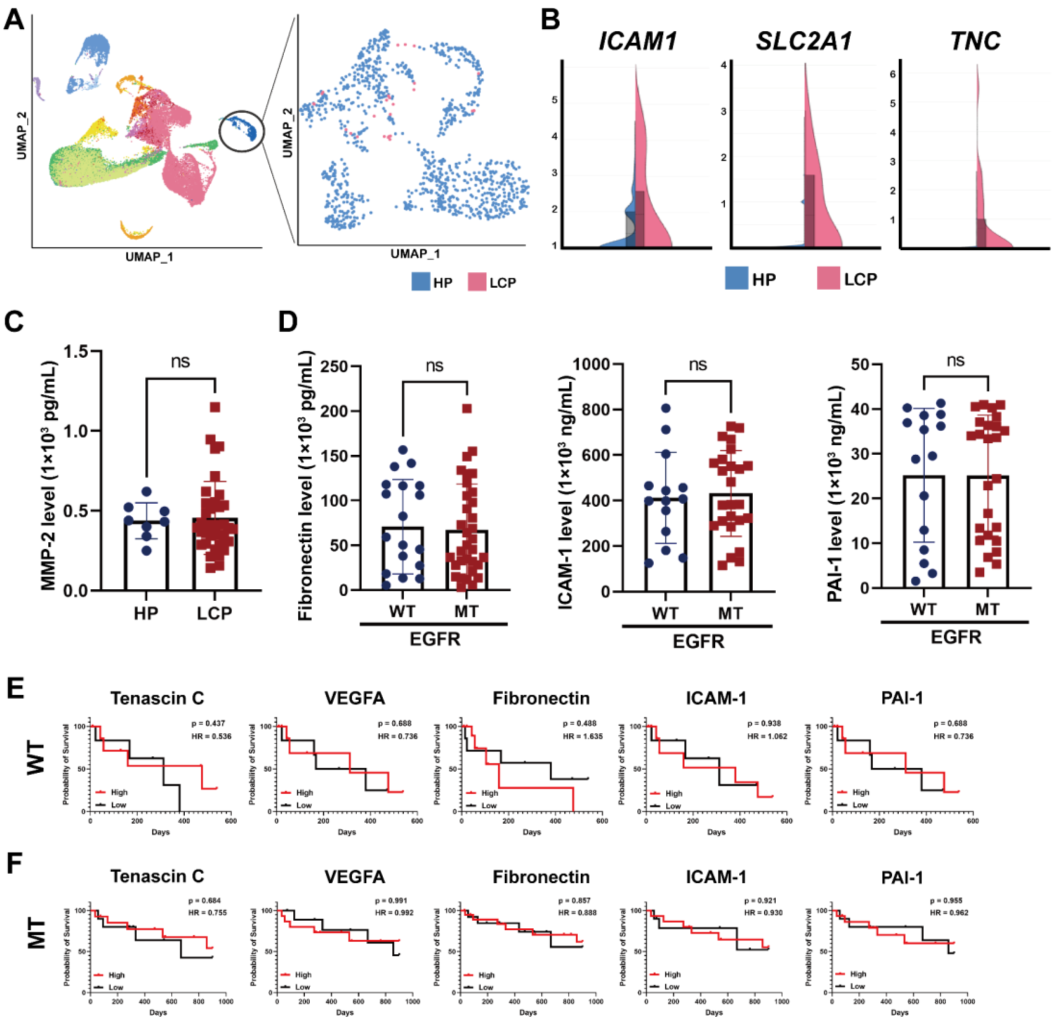

A
UMAP_2
UMAP_1
HP
LCP
B
ICAM1
SLC2A1
TNC
HP
LCP
C
MMP-2 level (1×10³ pg/mL)
ns
HP
LCP
D
Fibronectin level (1×10³ pg/mL)
ICAM-1 level (1×10³ ng/mL)
PAI-1 level (1×10³ ng/mL)
ns
WT
MT
EGFR
E
WT
Tenascin C
VEGFA
Fibronectin
ICAM-1
PAI-1
p = 0.437 HR = 0.536
p = 0.688 HR = 0.736
p = 0.488 HR = 1.635
p = 0.938 HR = 1.062
p = 0.688 HR = 0.736
F
MT
Tenascin C
VEGFA
Fibronectin
ICAM-1
PAI-1
p = 0.684 HR = 0.755
p = 0.991 HR = 0.992
p = 0.857 HR = 0.888
p = 0.921 HR = 0.930
p = 0.955 HR = 0.962
Probability of Survival
Days
High
Low

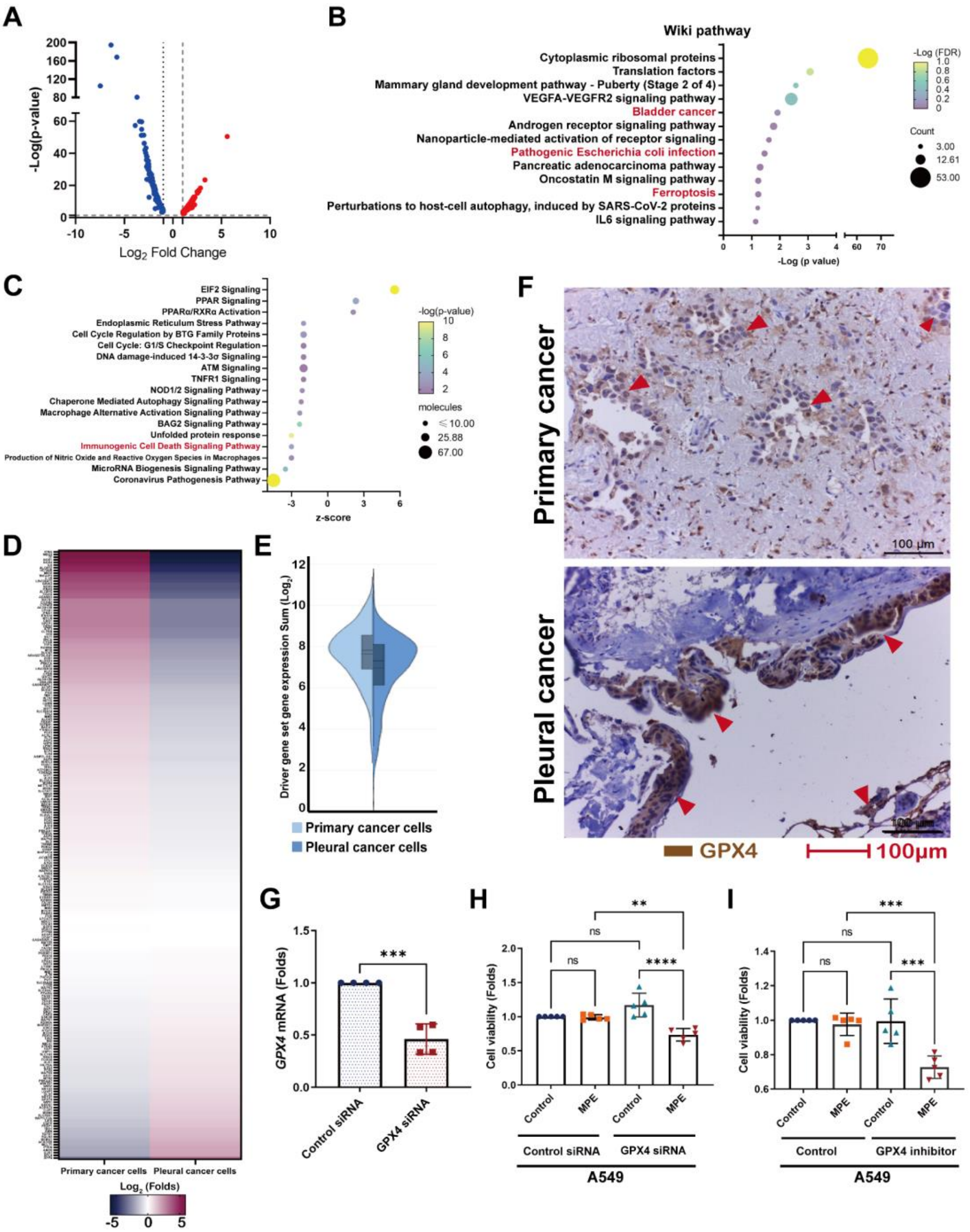
A
-Log(p-value)
Log2 Fold Change
B
Wiki pathway
Cytoplasmic ribosomal proteins
Translation factors
Mammary gland development pathway - Puberty (Stage 2 of 4)
VEGFA-VEGFR2 signaling pathway
Bladder cancer
Androgen receptor signaling pathway
Nanoparticle-mediated activation of receptor signaling
Pathogenic Escherichia coli infection
Pancreatic adenocarcinoma pathway
Oncostatin M signaling pathway
Ferroptosis
Perturbations to host-cell autophagy, induced by SARS-CoV-2 proteins
IL6 signaling pathway
-Log (FDR)
Count
-Log (p value)
C
EIF2 Signaling
PPAR Signaling
PPARα/RXRα Activation
Endoplasmic Reticulum Stress Pathway
Cell Cycle Regulation by BTG Family Proteins
Cell Cycle: G1/S Checkpoint Regulation
DNA damage-induced 14-3-3σ Signaling
ATM Signaling
TNFR1 Signaling
NOD1/2 Signaling Pathway
Chaperone Mediated Autophagy Signaling Pathway
Macrophage Alternative Activation Signaling Pathway
BAG2 Signaling Pathway
Unfolded protein response
Immunogenic Cell Death Signaling Pathway
Production of Nitric Oxide and Reactive Oxygen Species in Macrophages
MicroRNA Biogenesis Signaling Pathway
Coronavirus Pathogenesis Pathway
-log(p-value)
molecules
z-score
D
Primary cancer cells
Pleural cancer cells
Log2 (Folds)
E
Driver gene set gene expression Sum (Log2)
Primary cancer cells
Pleural cancer cells
F
Primary cancer
Pleural cancer
100 µm
GPX4
100µm
G
GPX4 mRNA (Folds)
Control siRNA
GPX4 siRNA
H
Cell viability (Folds)
Control
MPE
Control siRNA
GPX4 siRNA
A549
I
Cell viability (Folds)
Control
MPE
GPX4 inhibitor
A549

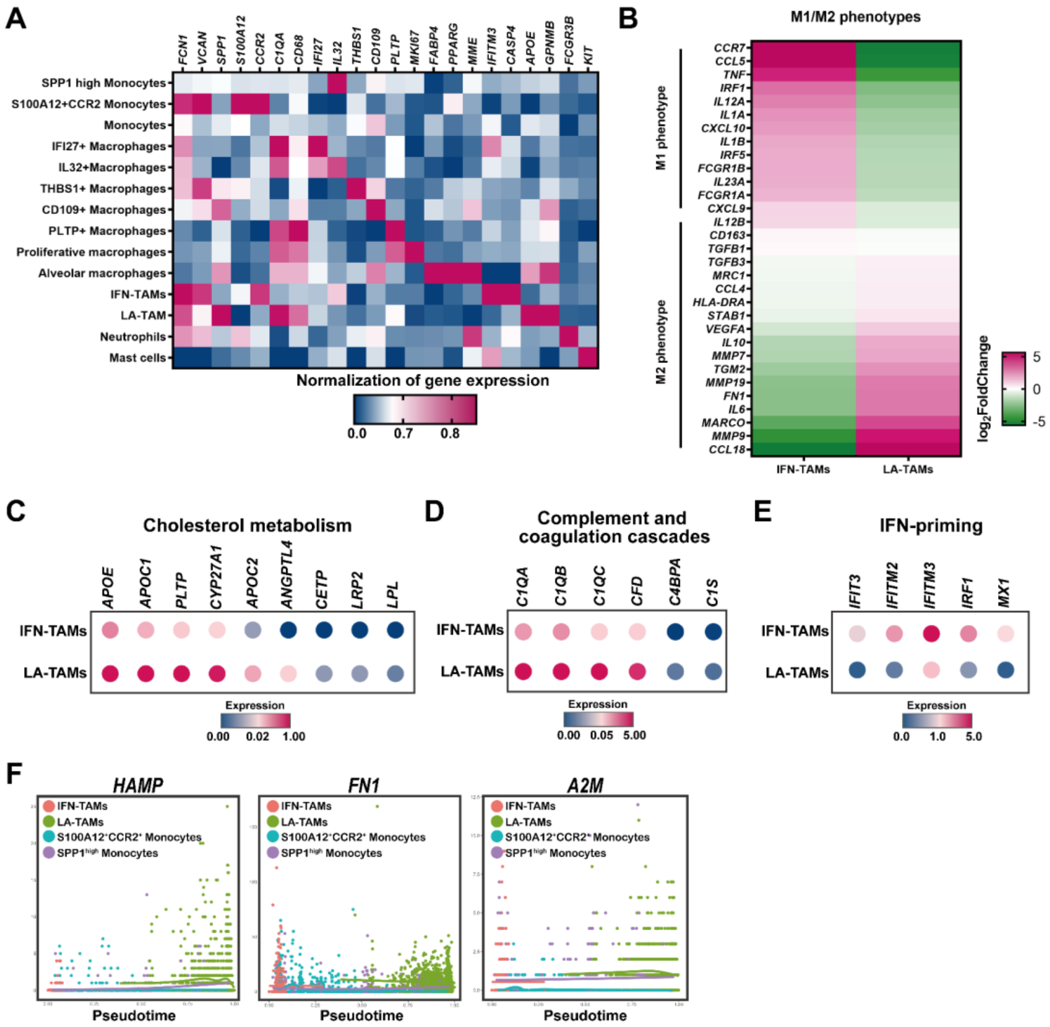

A
B
M1/M2 phenotypes
M1 phenotype
M2 phenotype
IFN-TAMs
LA-TAMs
log2FoldChange
Normalization of gene expression
C
Cholesterol metabolism
D
Complement and coagulation cascades
E
IFN-priming
Expression
F
HAMP
FN1
A2M
Pseudotime

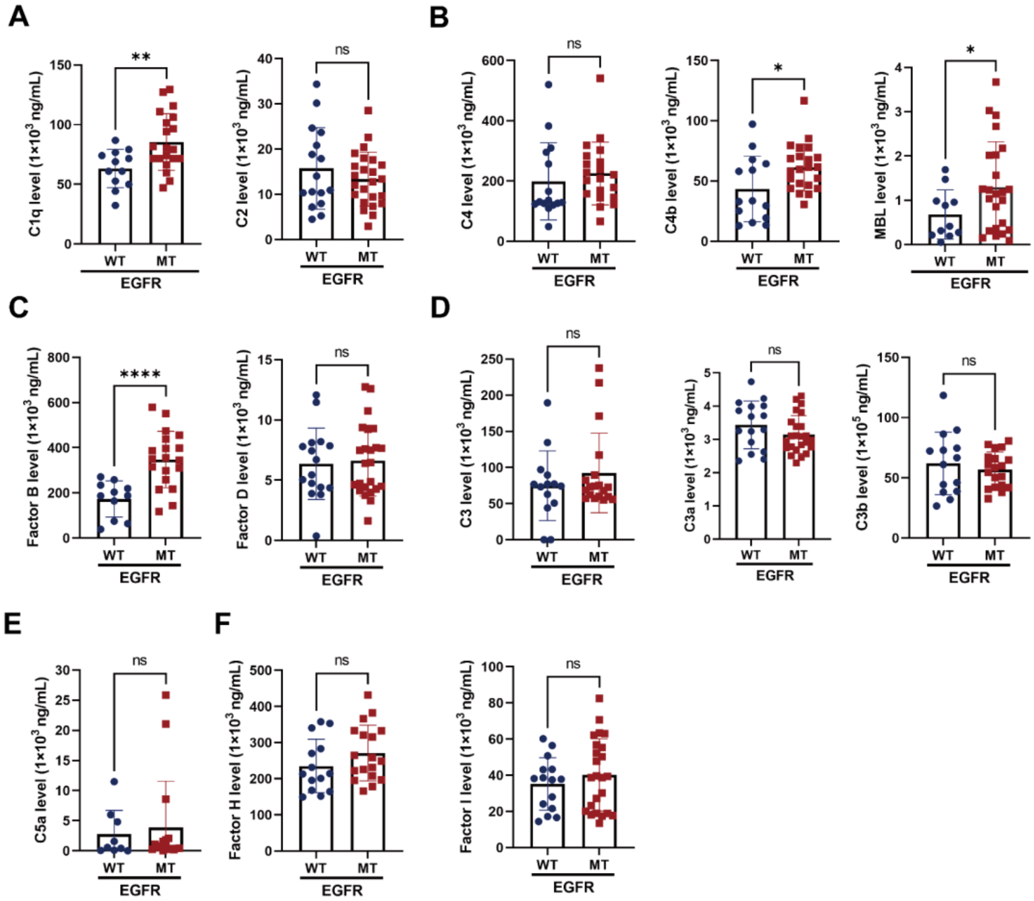
A
B
C
D
E
F
C1q level (1×10^3 ng/mL)
C2 level (1×10^3 ng/mL)
C4 level (1×10^3 ng/mL)
C4b level (1×10^3 ng/mL)
MBL level (1×10^3 ng/mL)
Factor B level (1×10^3 ng/mL)
Factor D level (1×10^3 ng/mL)
C3 level (1×10^3 ng/mL)
C3a level (1×10^3 ng/mL)
C3b level (1×10^5 ng/mL)
C5a level (1×10^3 ng/mL)
Factor H level (1×10^3 ng/mL)
Factor I level (1×10^3 ng/mL)
WT
MT
EGFR
**
*
****
ns

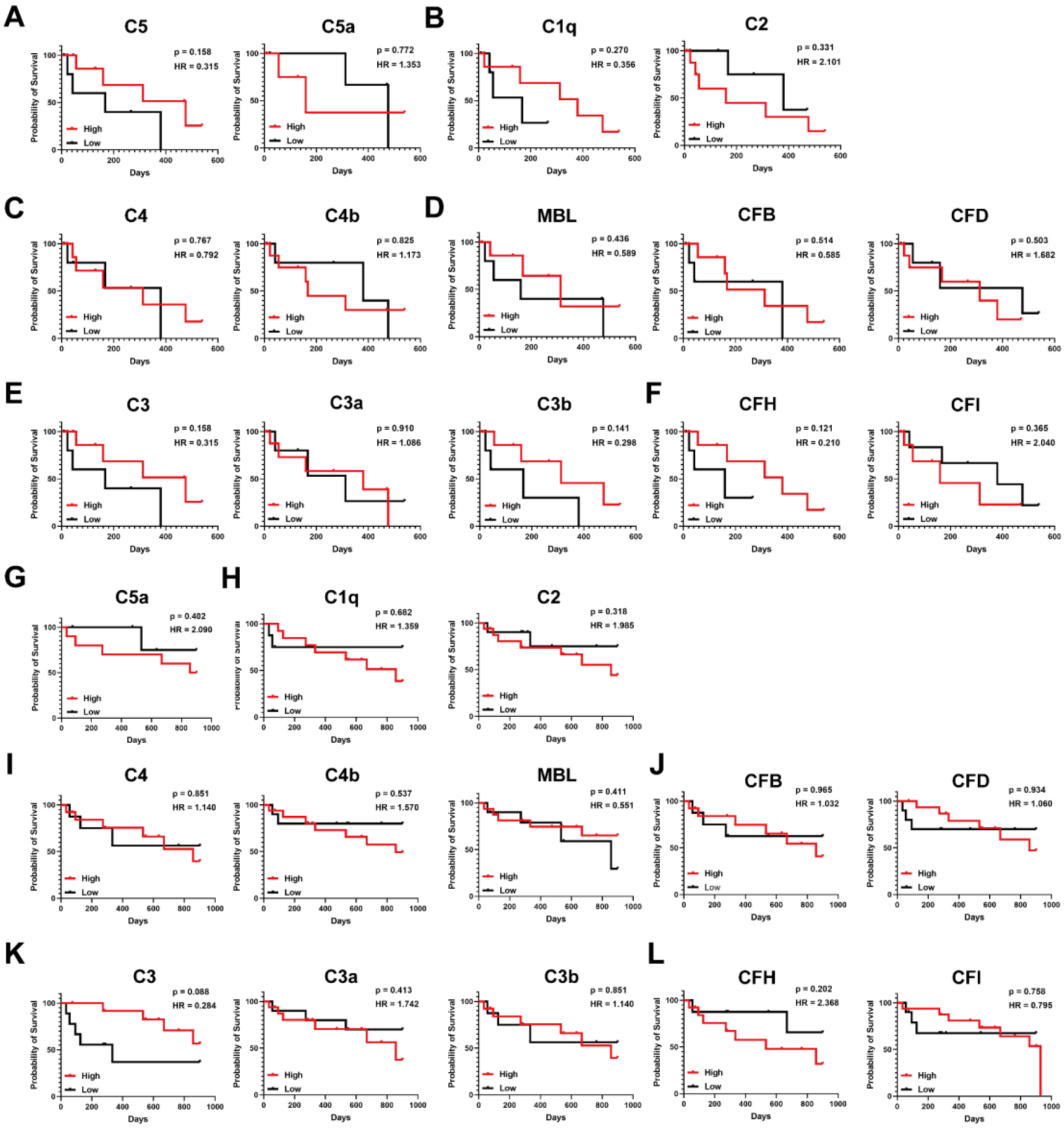
A
C5
p = 0.158
HR = 0.315
C5a
p = 0.772
HR = 1.353
B
C1q
p = 0.270
HR = 0.356
C2
p = 0.331
HR = 2.101
C
C4
p = 0.767
HR = 0.792
C4b
p = 0.825
HR = 1.173
D
MBL
p = 0.436
HR = 0.589
CFB
p = 0.514
HR = 0.585
CFD
p = 0.503
HR = 1.682
E
C3
p = 0.158
HR = 0.315
C3a
p = 0.910
HR = 1.086
C3b
p = 0.141
HR = 0.298
F
CFH
p = 0.121
HR = 0.210
CFI
p = 0.365
HR = 2.040
G
C5a
p = 0.402
HR = 2.090
H
C1q
p = 0.682
HR = 1.359
C2
p = 0.318
HR = 1.985
I
C4
p = 0.851
HR = 1.140
C4b
p = 0.537
HR = 1.570
MBL
p = 0.411
HR = 0.551
J
CFB
p = 0.965
HR = 1.032
CFD
p = 0.934
HR = 1.060
K
C3
p = 0.088
HR = 0.284
C3a
p = 0.413
HR = 1.742
C3b
p = 0.851
HR = 1.140
L
CFH
p = 0.202
HR = 2.368
CFI
p = 0.758
HR = 0.795
Probability of Survival
Days
High
Low

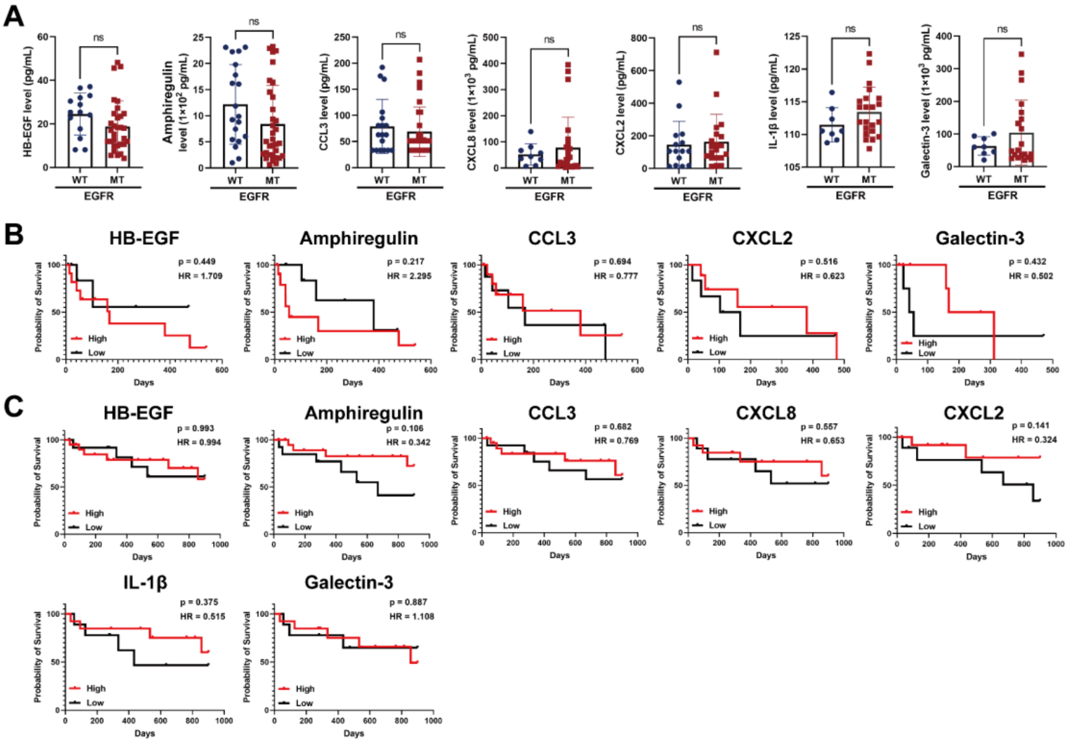
A
HB-EGF level (pg/mL)
Amphiregulin level (1×10[2] pg/mL)
CCL3 level (pg/mL)
CXCL8 level (1×10[3] pg/mL)
CXCL2 level (pg/mL)
IL-1β level (pg/mL)
Galectin-3 level (1×10[3] pg/mL)
ns
WT MT
EGFR
B
HB-EGF p = 0.449 HR = 1.709
Amphiregulin p = 0.217 HR = 2.295
CCL3 p = 0.694 HR = 0.777
CXCL2 p = 0.516 HR = 0.623
Galectin-3 p = 0.432 HR = 0.502
Probability of Survival
Days
High
Low
C
HB-EGF p = 0.993 HR = 0.994
Amphiregulin p = 0.106 HR = 0.342
CCL3 p = 0.682 HR = 0.769
CXCL8 p = 0.557 HR = 0.653
CXCL2 p = 0.141 HR = 0.324
IL-1β p = 0.375 HR = 0.515
Galectin-3 p = 0.887 HR = 1.108

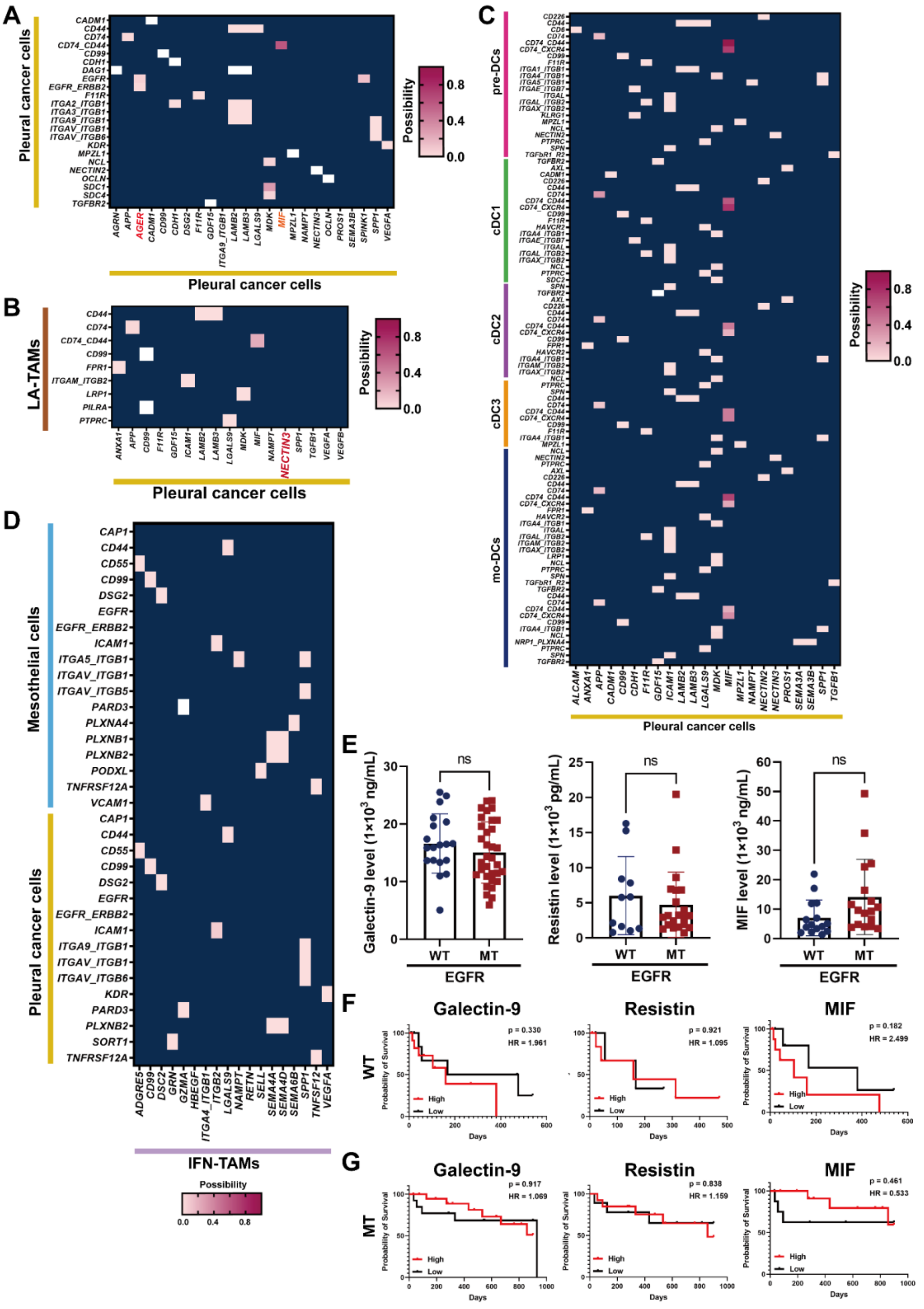

A
Pleural cancer cells
Possibility
0.8
0.4
0.0
B
LA-TAMs
C
pre-DCs
cDC1
cDC2
cDC3
mo-DCs
D
Mesothelial cells
IFN-TAMs
E
Galectin-9 level (1×10^3 ng/mL)
Resistin level (1×10^3 pg/mL)
MIF level (1×10^3 ng/mL)
ns
WT
MT
EGFR
F
Galectin-9
Resistin
MIF
p = 0.330
HR = 1.961
p = 0.921
HR = 1.095
p = 0.182
HR = 2.499
Probability of Survival
Days
High
Low
G
p = 0.917
HR = 1.069
p = 0.838
HR = 1.159
p = 0.461
HR = 0.533
