# Supplementary material for: Characterization of the pleural microenvironment niche and cancer transition using single-cell RNA sequencing in EGFR-mutated lung cancer
Source: Theranostics. 2023 Aug 6;13(13):4412–29. doi: 10.7150/thno.85084 (PMC10465223; doi:10.7150/thno.85084)
Supplement: Supplementary file 2 — Supplementary tables. [file thnov13p4412s2.zip › Supplementary Table/Table S3 The primers used in our study.docx]

Table S3. The primers used in our study

| Gene | Primers |
| --- | --- |
| GPX4 | F ccgtgtaaccagttcgggaa  R GCCCTTGGGTTGGATCTTCA |
| ACSL4 | F ggaatgacaggccagtgtga  R TGCTGGACTGGTCAGAGAGT |
| FTL | F caggcctcctacacctacct  R CCACTCATCTTCAGCTGGCT |
| NUPR1 | F GACTCCAGCCTGGATGAATCTG  R CTTCTCTCTTGGTGCGACCTTTC |
| GAPDH | F TTCACCACCATGGAGAAGGC  R GGCATGGACTGTGGTCATGA |

Abbreviations: GPX4, Glutathione Peroxidase 4; ACSL4, Acyl-CoA Synthetase Long Chain Family Member 4; FTL, Ferritin Light Chain; NUPR1, Nuclear Protein 1, Transcriptional Regulator; GAPDH, Glyceraldehyde-3-Phosphate Dehydrogenase.
