# Supplementary material for: Characterization of the pleural microenvironment niche and cancer transition using single-cell RNA sequencing in EGFR-mutated lung cancer
Source: Theranostics. 2023 Aug 6;13(13):4412–29. doi: 10.7150/thno.85084 (PMC10465223; doi:10.7150/thno.85084)
Supplement: Supplementary file 2 — Supplementary tables. [file thnov13p4412s2.zip › Supplementary Table/Table S9-Gene list of functional scores.docx]

Table S9. The gene sets for phagocytosis and APC

| Phagocytosis | APC |
| --- | --- |
| RAB5A | CD74 |
| RAB34 | TAP1 |
| RAB7A | TAP2 |
| RAB7B | TAPBP |
| CDC42 | CD38 |
| CLEC5A | HLA-DRA |
| CLEC7A | HLA-DOB |
| FPR1 | HLA-DRB5 |
| FPR3 | HLA-A |
| FPR2 | HLA-B |
| NLRP3 | HLA-C |
| CD33 |  |

Abbreviation: APC, Antigen-presenting cell.
